# Photoexcited and ground-state diradical(oid) character in a triquino[3]radialene†

**DOI:** 10.1039/d5sc01676b

**Published:** 2025-05-20

**Authors:** Bethany K. Hillier, Damon M. de Clercq, Stephen D. S. Bortolussi, Simona S. Capomolla, Michael P. Nielsen, Katarzyna Młodzikowska-Pieńko, Renana Gershoni-Poranne, Timothy W. Schmidt, Martin D. Peeks

**Affiliations:** a School of Chemistry, UNSW Sydney NSW 2052 Australia m.peeks@unsw.edu.au; b School of Photovoltaics and Renewable Energy Engineering, UNSW Sydney NSW 2052 Australia; c Schulich Faculty of Chemistry, Technion-Israel Institute of Technology Technion City Haifa 32000 Israel

## Abstract

The unusual [3]radialene motif at the centre of triquino[3]radialene permits exceptional electronic conjugation between the three quinone arms of the molecule, giving rise to a remarkably low-energy and intense absorption spectrum. Since triquino[3]radialene's initial synthesis in the 1970s, questions have been posed around the molecule's potential diradical(oid) character. In this work we present new synthetic approaches to triquino[3]radialene and a thorough spectroscopic and computational evaluation of its properties. Despite broad UV-visible absorption, the triquino[3]radialene is non-emissive. An investigation by transient absorption spectroscopy reveals that the initially-formed excited state of triquino[3]radialene decays to a triplet state with a lifetime of 0.82 μs. Using computational chemistry, we compare triquino[3]radialene to Yang's biradical and galvionxyl, with which it is structurally similar. DFT reveals insights into the role of aromaticity in the photoexcited triplet state, where quinoidal rings become more aromatic. MCSCF and CIPT2 calculations reproduce the experimental spectra and reveal a diradical character of *y* = 0.05 in triquino[3]radialene.

## Abstract 2

Triquino[3]radialene (R = *t*Bu) has a photoaccessible triplet state, assigned by TA spectroscopy and CIPT2 calculations. The ground- and excited-states have global magnetically-induced ring currents according to calculations.

## Graphical abstract

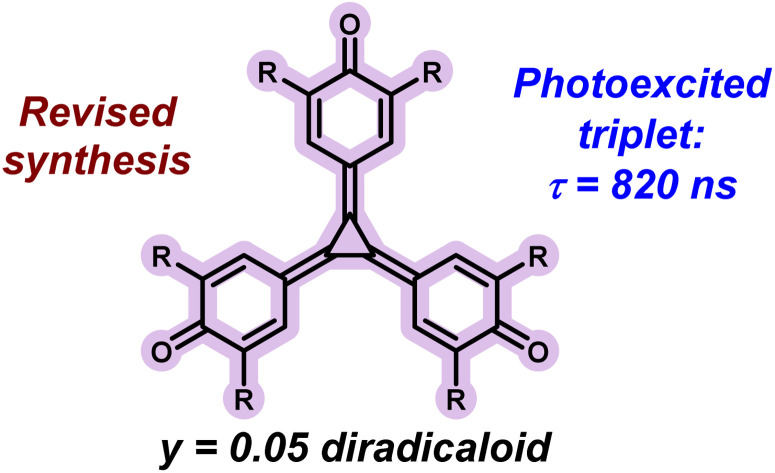

## Introduction

**Fig. 1:**
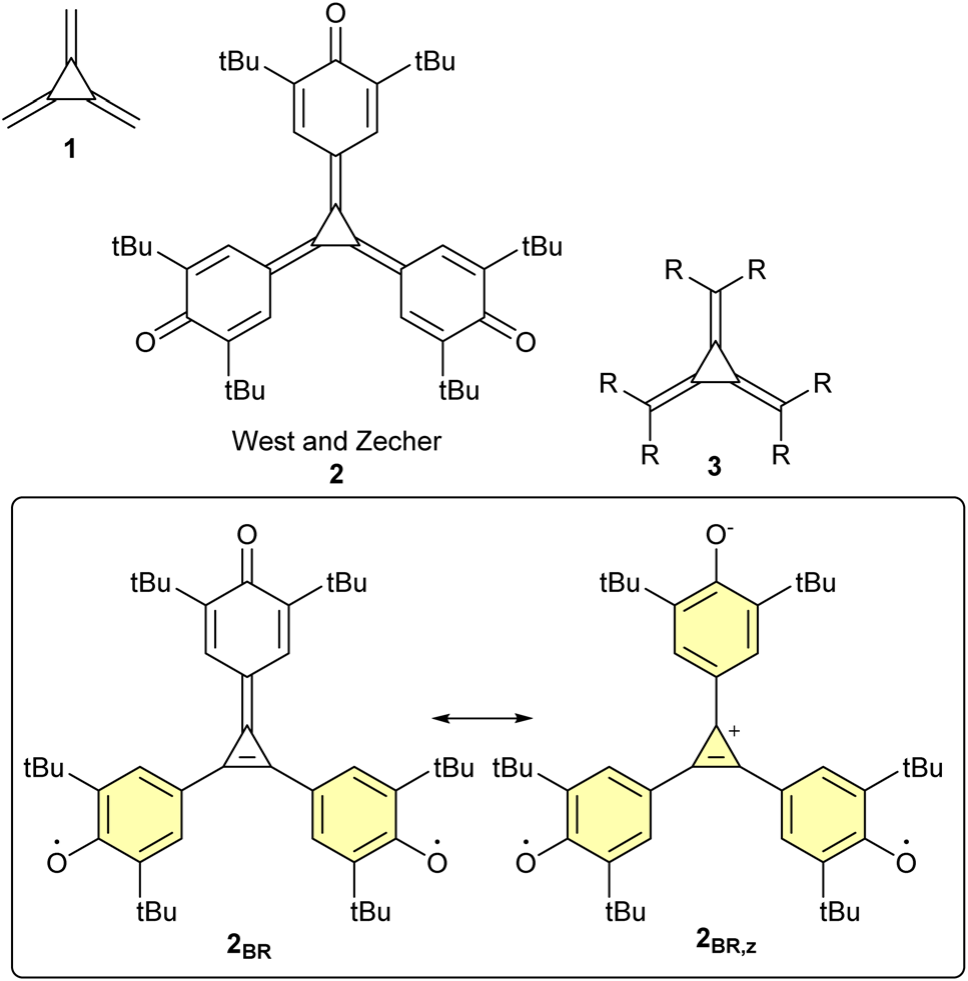
[3]Radialene and its selected analogues. In the lower box, cycles with [4*n* + 2] π-electrons are highlighted yellow.

[3]Radialene (C_6_H_6_, 1, Fig. 1) was first prepared by Dorko in 1965 (ref. 1) and is the smallest cyclic molecule capable of three-terminal π-conjugation. Comprising a central cyclopropane ring with three exocyclic methylene groups, it presents an unusual conjugation motif: every exocyclic position is linearly conjugated to the other two. On account of this character, it has been described as omni-conjugated^2^ and is the smallest cyclic omni-conjugated molecule. Although the parent [3]radialene is unstable, various substituted derivatives have been prepared, including triquino[3]radialenes (the subject of this article, 2),^3,4^ hexaaryl[3]radialenes (3, R = Ar),^5,6^ phospha[3]radialene,^7,8^ and a protected hexaethynyl[3]radialene (3, R = triisopropylsilylacetylene).^9^

**Fig. 2:**
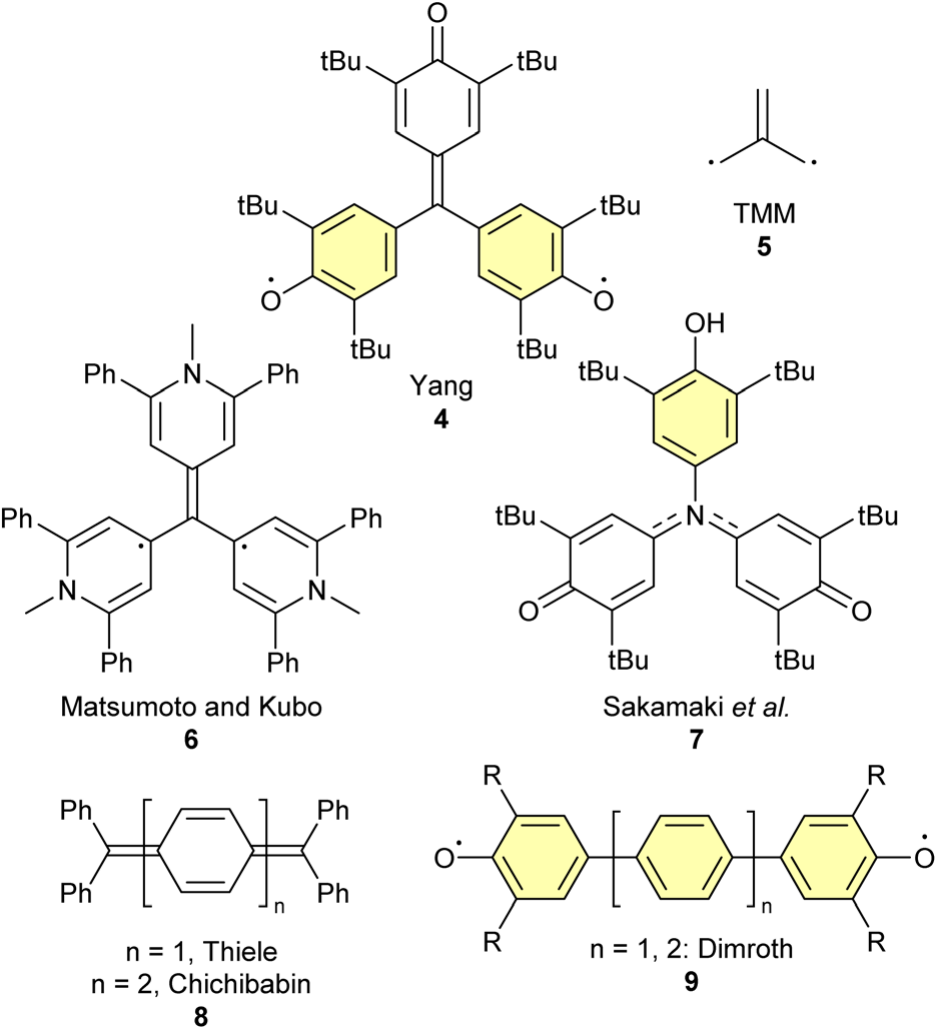
Examples of diradical and diradicaloid molecules. Aromatic sextets are shaded yellow. R = Ph.

In 2008, a series of [3]radialene derivatives were disclosed in the patent literature as materials for organic LEDs.^10^ More recently, substituted [3]radialenes have been reported as potential organic electrolyte materials for batteries.^11,12^ However, to our surprise, there has been no in-depth study of the effect of the omni-conjugated core on the electronic properties of π-extended [3]radialenes. Realizing that triquino[3]radialene can be analogized both to Yang's biradical 4 (and related molecules, Fig. 2) and to quinones, and that there was an opportunity to build upon studies by West and Zecher from the 1960 and 70s,^3,4,13^ we identified this molecule as an ideal subject for a comprehensive study. In the present work, we introduce a revised synthesis of triquino[3]radialene and a thorough study of its electronic properties through time-resolved spectroscopy and electronic structure calculations.

In their report of the synthesis of triquino[3]radialene in 1967, West and Zecher noted that the molecule has a strong (log *ε* = 4.7) and low-energy (extending to 770 nm) absorption spectrum.^3,13^ In a later paper, they revealed that they had expected the triquino[3]radialene to adopt a biradical state, but had seen little convincing evidence of such character by electron spin resonance (ESR).^4^ The only ESR signal in a sample of 2 was a doublet-type signal found at high temperatures in naphthalene, likely attributable to a decomposition product.^4^ The NMR spectrum of 2 is sharp and fully-assignable; all evidence suggests that the molecule has a closed-shell singlet ground-state.

The structure of 2 can be drawn in three different ways: as a closed-shell molecule, or as a diradical either with (2_BR,z_), or without (2_BR_), zwitterionic character (Fig. 1). Organic molecules which adopt diradical (or biradical) electronic states are of immense interest both conceptually and practically,^14,15^ yet open questions remain about the factors which drive adoption of diradical rather than closed-shell states. If a molecule gains aromaticity in its diradical state, then the diradical might be favored if the associated aromatic stabilization outweighs the corresponding loss of a covalent bond. Pro-aromatic molecules, of which quinones are an example, are described as having an “irresistible wish to be diradicals”.^15^ It is therefore surprising that 2 is not a diradical, given the potential gain of aromaticity in the diradical states: 2_BR_ gains two Clar aromatic rings^16^ and 2_BR,z_ gains four aromatic rings (of which three are Clar sextets, alongside a 2π cyclopropenium; aromatic rings are highlighted yellow in Fig. 1).

The diradical 2_BR_ bears a striking resemblance to Yang's biradical 4;^17^ the two molecules can be related by change of the [3]radialene core for a trimethylenemethane (TMM, 5, see Fig. 2). In terms of resonance structures, both 4 and the hypothetical 2_BR_ benefit from the aromatic stabilization afforded by the formation of two Clar sextets. Nevertheless, the exact extent of this stabilization is unknown, which makes it difficult to attribute the former's high-spin ground state to aromaticity. Instead, the most compelling explanation comes from 4's non-Kekulé structure: it is impossible to draw a plausible structure in which all electrons are paired. Yang's biradical was reported to be fully delocalized based on room-temperature EPR spectroscopy.^18^ While the spectrum resolves at low temperature,^18,19^ a crystallographic and computational study by Bock *et al.* argued for a fully-symmetric, fully-delocalized triplet biradical structure.^20^ The kinetic stability of Yang's biradical can be rationalized by the steric blocking effect of the *tert*-butyl groups. The *tert*-butyl groups block the phenoxy radicals from abstracting hydrogen from solvent molecules upon photoexcitation, which would otherwise result in closed-shell phenol products.^21^ Derivatives of Yang's biradical have been prepared: a pyridinyl derivative 6 was reported by Kubo and co-workers to adopt a ground-state triplet.^22^ However, when the central carbon atom of 4 was replaced with nitrogen, as in 7, the molecule had a closed-shell electronic structure, despite the facts that a diradical form can be drawn with two aromatic sextets and that no convincing Kekulé structure can be drawn.^23^

The emerging picture is that diradical character is difficult to predict and that, even given heuristics such as aromaticity and non-Kekulé structure, exceptions still arise. This tension between aromaticity and diradical character is further illustrated by Thiele's and Chichibabin's hydrocarbons 8,^24,25^ and their phenol/quinone analogues. Both Thiele's and Chichibabin's molecules are singlets, and the latter has appreciable (∼60%) diradical character.^26^ The quinone analogues of Thiele's and Chichibabin's hydrocarbons are both singlets,^27^ but have a rich photochemistry that relies on their photogenerated triplet states.^28–30^ Dimroth and co-workers reported that quinones with further π-extension, as in 9, had strong EPR signals.^31^ Later studies (where R = Ph in Dimroth's terphenoquinone 9 was replaced with R = *t*Bu) showed no sign of a triplet ground state,^32,33^ but spectroscopic and crystallographic data supported the presence of some diradicaloid character in the singlet ground state.^33^

Each of the molecules described in the preceding section has either linear conjugation or conjugation through a central TMM core. [3]Radialene's unique tripodal pro-quinoidal conjugation presents a fundamentally distinct form of connection, and the purpose of the present article is to evaluate the effect of this conjugation motif through the lens of past research on Yang's biradical and extended quinones. As we will show, triquino[3]radialene has a singlet ground state with 5% diradicaloid character according to MCSCF calculations, and we observe a low-lying long-lived triplet state by time-resolved spectroscopy. While the MCSCF wavefunction methods perform very well for reproducing the experimental properties, density functional theory methods fare less well, illustrating the particular challenges associated with studying molecules which have degenerate states and diradicaloid character. The remainder of this article will first describe revisions to West and Zecher's synthesis of triquino[3]radialene, followed by our spectroscopic and computational results.

## Results and discussion

### Synthesis

**Scheme 1:**
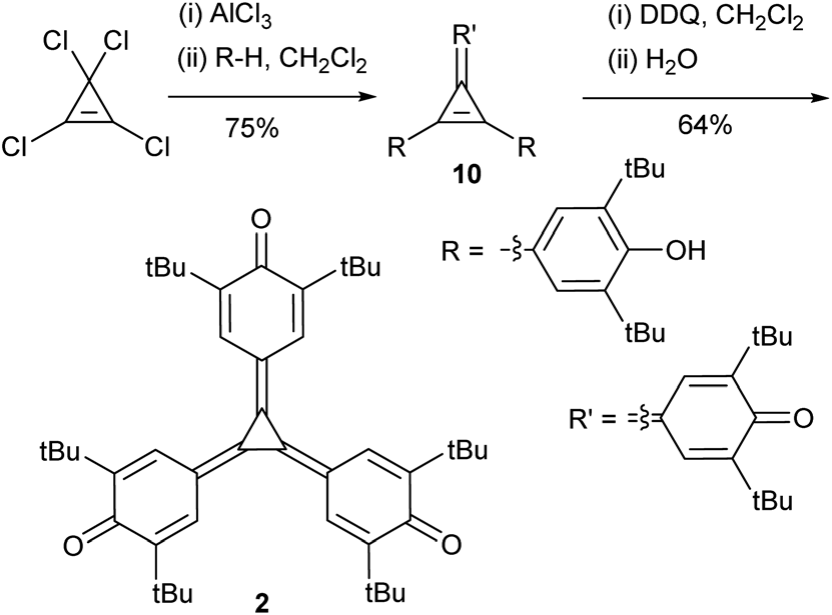
Revised synthesis of triquino[3]radialene, which substitutes benzene and PbO_2_ in the original for CH_2_Cl_2_ and DDQ, respectively.

West and Zecher's original synthesis of triquino[3]radialenes involved Friedel–Crafts alkylation of a suitable phenol derivative with tetrachlorocyclopropene, affording a mixture of the diarylquinocyclopropene 10 and the related triarylcyclopropenium chloride, followed by oxidation with PbO_2_ or aqueous basic K_3_[Fe(CN)_6_] in benzene to afford the triquino[3]radialene.^3^ We followed the same overall logic, but sought to eliminate benzene and lead from the synthesis in order to develop a safer and greener process, and due to the increasing regulatory burden associated with these reagents. The initial Friedel–Crafts reaction proceeded without challenge, though we isolated solely the diarylquinocyclopropene 10 (confirmed by X-ray crystallography, ESI Fig. S41†). We were able to generate the cyclopropenium chloride by treating the diarylquinocyclopropene 10 with acid (ESI Scheme S2†). Its oxidation in a biphasic reaction mixture using aqueous basic K_3_[Fe(CN)_6_] was successful where the organic solvent was either dichloromethane or α,α,α-trifluorotoluene, but in relatively low yields (43% and 20% respectively, ESI Table S1†). We decided to explore homogeneous reaction conditions using an organic-phase two-electron oxidant. Chloranil was capable of producing the target triquino[3]radialene 2 in dichloromethane, and 2,3-dichloro-5,6-dicyano-1,4-benzoquinone (DDQ) was competent for the reaction in dichloromethane, α,α,α-trifluorotoluene, and acetonitrile. Our revised synthetic procedure is shown in Scheme 1 and the yields of different oxidation conditions are provided in ESI Table S1.†

Consistent with West and Zecher's reports, we found that steric bulk at the 2,6-positions of the starting phenols was essential to the success of the final oxidation.^3^ Oxidation of the methyl-substituted analogue leads to a short-lived colour change – consistent with formation of radialene – before the colour returns to that of the cyclopropenium, which is confirmed by NMR spectroscopy. Reflecting on the similar reactivity of unprotected phenoxy radicals,^21^ this result indicates that the triquino[3]radialene has some diradicaloid character. The aryl protons of 2 resonate at 7.57 ppm, intermediate between the chemical shifts of the aryl protons in 3,3′,5,5′-tetra-*tert*-butyl-4,4′-diphenoquinone (7.71 ppm)^27^ and its analogous diphenol (7.30 ppm).^34,35^ By way of further comparison, the protons in *p*-benzoquinone have a chemical shift of 6.78 ppm. The IR spectra of 10 and 2 are provided in ESI Fig. S29 and S30† respectively. The diarylquinocyclopropene has two bands in the carbonyl region: 1807 cm^−1^ (weak, attributable to the cyclopropane C=C stretch)^4^ and 1591 cm^−1^ (medium). The triquino[3]radialene product 2 has just one C=O stretch at 1588 cm^−1^ (very strong).

### Optical spectroscopy

The diarylquinocyclopropene 10 is deep-orange coloured with an absorption cut-off around 550 nm, and a major peak at 410 nm (log *ε* = 4.879) (ESI Fig. S23†). The extended electronic delocalization of the triquino[3]radialene 2 is illustrated by the emergence of a strong absorption band between 550 nm and 850 nm, comprising two distinct peaks (686 nm and 767 nm) and two shoulders (centred around 565 nm and at 629 nm). The absorption spectrum of the triquino[3]radialene did not change as a function of solvent polarity as up to 20% methanol was titrated into CH_2_Cl_2_ (ESI Fig. S30†), suggesting that there is no charge-transfer character and supporting an assignment of the absorption spectrum as being dominated by π–π* transitions. We observed no emission from the triquino[3]radialene in the range 300 nm to 1600 nm. We measured the NIR absorption spectrum up to 1600 nm but observed no weak transitions which might have been attributable to double-excitations reflective of the molecule's diradical character.

**Fig. 3:**
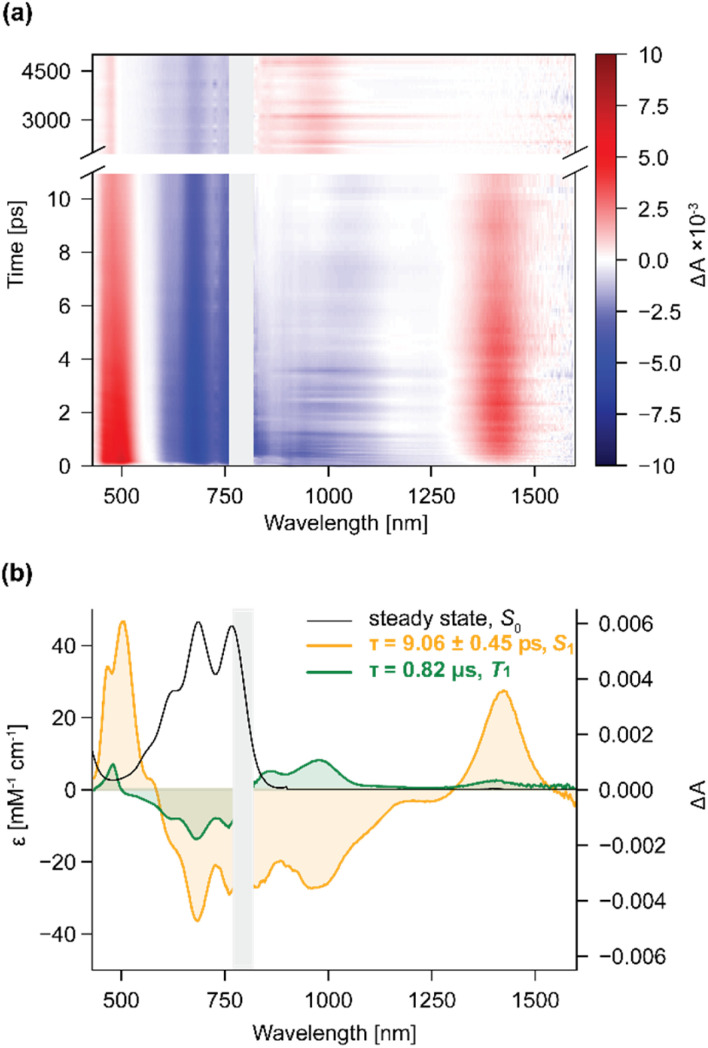
(a) Transient absorption of 2 after excitation at 800 nm; (b) ground-state spectrum of 2 (black) and decay-associated spectra of the short-lived species S_1_ (orange) and the longer-lived species T_1_ (green).

Transient absorption spectroscopy of solutions of 2 following excitation at 800 nm revealed the initial formation of a species with a lifetime of 9 ps. This species has a ground state bleach (GSB) that extends from 520–820 nm. The GSB is accompanied by excited state absorptions (ESA) in the range of 460–510 nm and at 1423 nm, which we assign to the S_1_ state as they decay at the same rate as the stimulated emission (860–1250 nm) (Fig. 3a). The S_1_ spectrum decays producing a simplified and narrowed ESA at 474 nm, and a broad ESA at 870 and 970 nm (Fig. 3a and ESI Fig. S9†). Longtime TA spectroscopy probing the regions between 430-760 nm showed that the shifted ESA at 474 nm decayed with lifetime 0.82 μs, hence we assign these long-lived signals as a triplet (T_1_) spectrum. The lifetimes were determined either from direct fitting of a single exponential, or from a sequential model; both approaches gave similar results (ESI Tables S2, S3 and Fig. S3–S8†). The decay-associated species are shown in Fig. 3b.

The excited-state absorption bands of the short-lived species do not change with solvent (CH_2_Cl_2_, toluene, and 2–20% MeOH in CH_2_Cl_2_, ESI Fig. S1†), however the *λ*_max_ of the stimulated emission is redshifted by 51 nm in toluene. The longer-lived species exhibits some subtle changes in its absorption spectrum: the peak at 970 nm in CH_2_Cl_2_ is redshifted by 34 nm in toluene, and progressively blue-shifted by a few nm as 2–20% of MeOH are added to CH_2_Cl_2_ (ESI Fig. S2†).

### Computational chemistry

To further probe the electronic structure of triquino[3]radialene 2, we performed density functional theory (DFT) and multi-reference wavefunction-based calculations. The wavefunction methods (MCSCF and CIPT2) proved more suitable for simulating the electronic states of 2 and provided results which agreed with the spectroscopic data. DFT was primarily used to evaluate the aromaticity of 2. To this end, we optimized 2 in both its singlet (S_0_) and triplet (T_1_) multiplicities at the CAM-B3LYP/6-31G* level, as implemented in Gaussian16,^36–40^ and investigated its aromatic character in these states using structural, magnetic, and electronic criteria. Optimized geometries were confirmed as minima by the absence of imaginary frequencies in their frequency calculations.

**Fig. 4:**
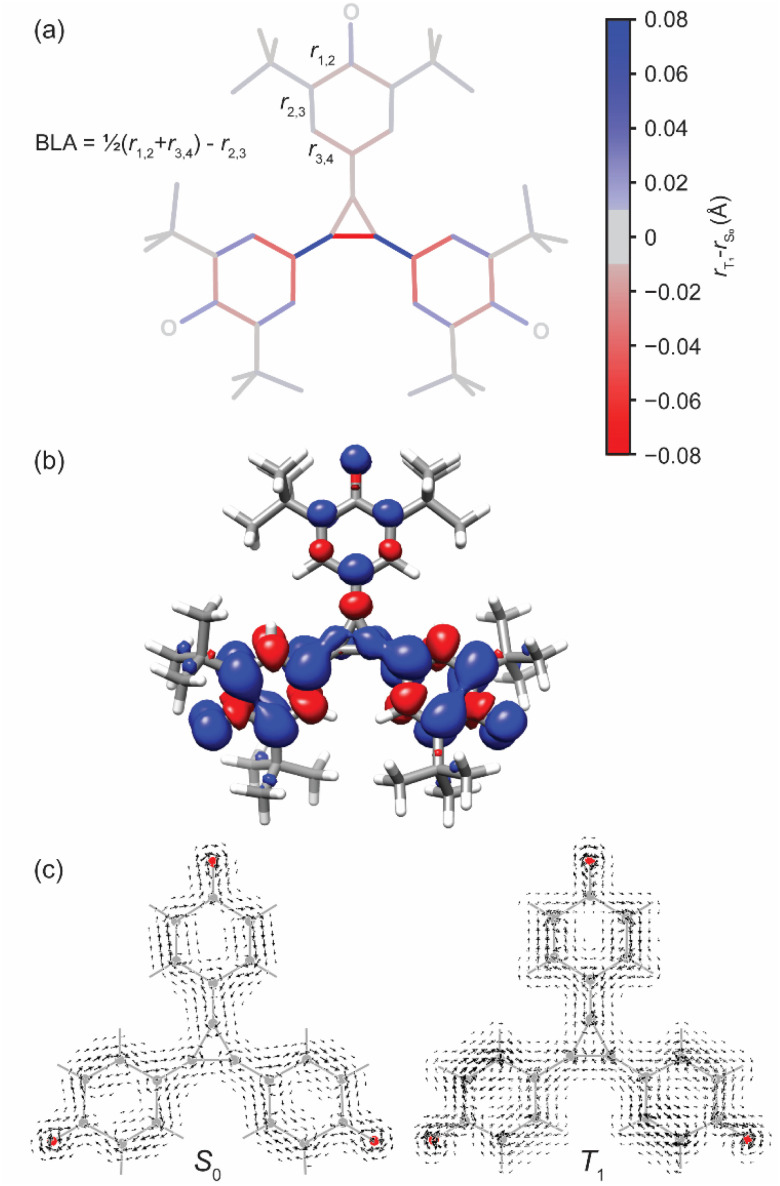
(a) Difference in bond lengths between triplet and singlet geometries (triplet–singlet); (b) spin density (CAM-B3LYP/6-31G*), isosurface plotted at 0.002; (c) magnetically-induced current densities of a truncated model of 2 (*t*Bu replaced with H), calculated 1.7 Å above the plane using SYSMOIC.^50^

The DFT-optimized geometry of the S_0_ state shows approximate three-fold symmetry, with an equilateral central cyclopropane core (*r*_C–C_ = 1.425 Å) and identical pendant six-membered (benzene) rings. Compared to benzene, the six-membered rings display reduced symmetry, evident in appreciable bond length alternation (BLA = 0.116 Å, see Fig. 4a for definition of BLA) and adopt a quinoidal distortion. These changes are indicative of decreased aromatic character, which can be assessed quantitatively with various metrics. The harmonic oscillator model of aromaticity (HOMA) assesses aromatic character based on molecular geometry.^41^ By definition, benzene has a HOMA value of 1.0; values close to zero (or below) correspond to non- or anti-aromaticity. The weaker aromatic character of the six-membered rings in 2 is evident in the HOMA values (HOMA = −0.294; Table 1). Negative HOMA values are relatively unusual, and have been reported for highly-substituted C_6_O_6_^6−^ and its salts,^42^ and for [6]radialene derivatives.^43,44^ In addition, we calculated nucleus independent chemical shifts (NICS),^45,46^ which measure the magnetic shielding at a point in space around a molecule. Negative NICS values correspond to aromatic (diatropic) ring currents; positive values correspond to antiaromatic (paratropic) ring currents, and values near zero correspond to non-aromaticity. In this work, we used the NICS(1.7)_*zz*_ metric – *i.e.* the *zz* component of the shielding tensor, which is most sensitive to aromaticity, at a point 1.7 Å above the center of each ring. This height has been recommended as an optimal balance between capturing the behavior of the π-orbitals and avoiding undesired contributions of σ-orbitals to the NICS value.^47^ Our results show that the six-membered rings in the S_0_ state of 2 are essentially non-aromatic, with NICS(1.7)_*zz*_ = −4.4 ppm, and the central ring is weakly aromatic, with NICS(1.7)_*zz*_ = −5.9 ppm (for comparison, at the same level of theory, NICS(1.7)_*zz*_ = ∼−20 ppm for benzene).^48,49^ We visualized the magnetically-induced current density in 2, and in a planar model of 2, using SYSMOIC (Fig. 4c and ESI Section 3.4†).^50^ The planar model, in which *t*Bu groups are replaced with H, allows us to separate the σ- and π-orbital contributions to the current density. The π-only currents are visualized as arrows in Fig. 4c, and reveal that there is a weak global diatropic current encompassing the entire molecule in the singlet state (S_0_). Local olefinic-type circulations are apparent around several bonds. The induced currents arising from all electrons are shown in ESI Fig. S14 and S15.† The molecule sustains ring currents with apparently “global” character: these currents traverse each of the pendant groups and circulate around the central radialene. Arrows attributable to atomic circulations are also visible around the *tert*-butyl groups and oxygen atoms. The anisotropy of the induced current density (ACID)^51,52^ and induced current density, shown in ESI Fig. S17 and S18,† provide a consistent picture of the ring currents in 2.

**Table 1:** Metrics of aromaticity in 2 and related molecules. Level of theory: CAM-B3LYP/6-31G*. NICS calculated at the centers of the 6-membered rings. See ESI Section 3.3 for further aromaticity indicators

		BLA (Å)	HOMA	NICS(1.7)_*zz*_ (ppm)
Galvinoxyl		0.100	0.092	−6.7, −7.4
Yang's biradical 4^a^		0.086	0.249	−7.5
Yang's biradical (xtal)		0.065 ± 0.006^b^	0.33 ± 0.05^b^	N/A
2 S_0_	Quinoid	0.116	−0.294	−4.4
	C_3_	N/A	N/A	−5.9
2 T_1_	Quinoid	0.107	−0.100	−6.9
	Benzenoid	0.068	0.277	−6.8, −7.0
	C_3_	N/A	N/A	−4.5

a *t*Bu groups were truncated to Me.

b Errors based on precision in reported crystallographic data (*i.e.* pm precision).

In its T_1_ state, 2 exhibits a reduction in symmetry from three-fold to two-fold, which is accompanied by several structural changes. The central three-membered ring takes on cyclopropene-like character (*r*_C=C_ = 1.347 Å; *r*_C–C_ = 1.418 Å), with a correspondingly shorter C=C bond to one of the pendant rings compared with that to the other two. In addition, the first ring displays a different geometry than the latter two, which are identical. These changes already indicate dominance of the 2_BR_ resonance structure over 2_BR,z_. As illustrated in Fig. 4a, the quinoidal structure of the two identical rings is reduced (*r*_2,3_ lengthens and *r*_1,2_/*r*_3,4_ shorten) while the change in the third ring is negligible, leading us to term the rings ‘benzenoid’ and ‘quinoidal’ (though these terms are relative, and none of them attain the aromatic character of benzene). All three rings show an increase in aromatic character relative to the singlet state, but the extent is not identical in all metrics. Structurally, the benzenoid rings show a greater increase in aromatic character (BLA = 0.068 Å, HOMA = 0.277) than the quinoidal ring (BLA = 0.107 Å, HOMA = −0.100). We found similar results using the HOMER method, which was designed for use in molecules with excited-state (anti)aromaticity (ESI Table S6†).^53^ In contrast, all three rings show similar magnetic aromaticity, with values of NICS(1.7)_*zz*_ = ∼−6.9 ppm. The current density plot in Fig. 4c suggests that the global diatropic current remains intact, co-existing with local olefinic currents.

The discrepancies between the metrics highlight the difficulty of assigning aromatic character in borderline cases. For this molecule, it is important to recognize that the olefinic currents in each C=C bond contribute to the NICS(1.7)_*zz*_ values, making it difficult to determine the extent to which each NICS value describes aromaticity, rather than local currents. Metrics of aromaticity which aim to quantify the extent of electronic delocalization in a ring are reported in ESI Section S3.3:† the FLU,^54^ PDI,^55^ I_ring_,^56^ and MCI^57^ values, calculated using AIMAll,^58^ and ESI-3D,^59,60^ are consistent with the HOMA results in Table 1: indicators of aromaticity increase in the triplet state, and the greatest increase is found in the benzenoid six-membered rings of 2_BR_. However, the HOMA metric and these delocalization metrics are not yet validated for molecules in their excited states, and so could offer artifactual results.

Further insight into the aromatic character may be found from the DFT spin density distribution. According to Baird's rule, triplet state benzene should have antiaromatic character.^61^ Surprisingly, we observe that the spin density (Fig. 4b) is mostly located on the benzenoid rings (see also ESI Fig. S20d†), which are predicted by HOMA and the electron delocalization metrics to be the rings with stronger aromatic character, and by the magnetic metrics to have similar aromaticity to the quinoidal ring. The fact that the presence of spin does not create antiaromatic character or even disrupt the (admittedly weak) aromatic character indicates that the aromaticity is not Baird-type and is not local. The existence of weak Hückel-aromatic character may be attributed to the delocalization of the spin over a large part of the molecule, or to partial localization of the unpaired electrons onto the phenoxyl oxygen. Natural resonance theory calculations using NBO7 (ref. 62) revealed that the resonance structures in which the radicals were centered on the two O atoms (2_BR_) bore a weight of 52% for the alpha-electrons of T_1_ (see ESI Fig. S20†).

We explored the aromaticity in 2 further by comparing calculated metrics of aromaticity for 2 with those for Yang's biradical 4 and the structurally similar monoradical, galvinoxyl. As detailed in Table 1, in galvinoxyl, both rings display weak aromatic character (HOMA = 0.092, NICS(1.7)_*zz*_ = −7 ppm). In contrast to 2_BR_, Yang's biradical retains its three-fold symmetry. The fact that the spin density in Yang's biradical is delocalized over three rings allows a rough evaluation of the impact of extending delocalization from two to three rings. The aromaticity metrics for the six-membered rings in Yang's biradical (HOMA = 0.249, NICS(1.7)_*zz*_ = −7.5 ppm) are slightly increased in comparison with 2_BR_ (HOMA = 0.277, NICS(1.7)_*zz*_ = ∼−6.9 ppm), in which the spin is delocalized over only two rings. This result supports the idea that delocalization of the spin enables retention of some Hückel aromatic character.

Overall, the results lend themselves to three main conclusions: (a) all six-membered rings appear to have slightly greater aromatic character in T_1_ than in S_0_ but can still be characterized as only weakly aromatic, most likely due to a global diatropic current; (b) there is no evidence of strong local aromaticity in the pendant rings or in the central C_3_ ring, supporting the notion that the 2_BR_ resonance structure is dominant. The 2_BR,z_ structure, which would contain four Hückel-aromatic rings (3 × 6π, 1 × 2π) is, at best, weakly contributing. This conclusion is unchanged with inclusion of a solvent model in the calculations, or with using B3LYP rather than CAM-B3LYP; (c) despite the presence of unpaired electrons, 2_BR_ exhibits Hückel-type aromaticity, rather than Baird-type antiaromaticity. The delocalization of the unpaired electrons over a large part of the molecule helps to mitigate their effect on the aromatic character.

A TD-DFT simulation of the singlet absorption spectrum using CAM-B3LYP dramatically over-estimated the energy of the lowest absorption, whereas the B3LYP^63,64^ functional afforded a closer match to experiment (ESI Fig. S21†). Given the apparent sensitivity of this structure to the choice of density functional approximation, we turned to higher-level wavefunction-based methods using Molpro.^65–67^ A CIPT2/cc-pVDZ calculation^68,69^ starting from a planar singlet geometry (with *t*Bu groups truncated to –H, symmetry *D*_3h_, calculation used *C*_2v_) reveals a lowest energy singlet excited state S_1_ (E′) at 695 nm, with T_1_ (also E′) appearing at 1207 nm, and T_2_ at 516 nm. The strong absorbance in the ground state at around 686 and 767 nm is assigned to S_1_ (calculated 695 nm); S_1_'s lowest-energy excited-state absorption, observed at 1423 nm, to S_2_ (calculated at 929 nm from S_1_ using [10,10] XMCQDPT2/cc-pVTZ). T_1_'s lowest-energy excited-state absorption at 970 nm closely matches the adiabatic energy of T_2_ (calculated 900 nm). Tabulated energies are available in the ESI, Tables S4 and S5.† We also estimated the diradicaloid character (*y*) of 2 from this series of calculations. Diradicaloid character can be quantified using many techniques, but perhaps the most conceptually simple rely on either the occupation number of the lowest unoccupied natural orbital (*n*_LUNO_), usually from a two-electrons-in-two-orbitals calculation (2o2e), or the coefficient of the double-excitation in the CI expansion (*c*_D_):^70^*y* = *n*_LUNO_ = 2*c*_D_^2^

Triquino[3]radialene 2 has a degenerate HOMO and so a 3o4e active space is required, yielding *y* = 0.05. By way of comparison, a perfectly closed-shell molecule has *y* = 0 and a perfect biradical (like Yang's biradical 4) would have *y* = 1.

## Conclusions

Photoexcitation of a triquino[3]radialene results in the generation of a long-lived (0.82 μs) state, which we assign with the help of computational chemistry as the triplet state. Calculations on this photoexcited diradical state suggest that Hückel aromaticity develops in the parts of the molecule on which the spin is delocalized, reflected in changes to the geometry of the affected six-membered rings. Surprisingly, there is no computational evidence of a zwitterionic form of central cyclopropene-quinone, which would afford two additional Hückel aromatic cycles.

The triquino[3]radialene can be viewed as a core-expanded analogue of Yang's biradical, in which the central carbon has been replaced with a cyclopropene group. The substitution can be considered a one-carbon extension of each arm of the tripodal chromophore, yet it has a dramatic effect on the properties of the molecules. Yang's molecule is a ground-state triplet in which the spin is fully delocalized. In contrast, the larger triquino[3]radialene is a ground-state singlet with approximately 5% diradical character, according to MCSCF calculations, which are in good agreement with the time-resolved spectroscopic data. Its photoexcited triplet is, according to DFT, localized on just two phenoxyl fragments. The results of our study illustrate a “tipping point” between localized and delocalized triplet structures, and open possibilities for the exploitation of other triquino[3]radialene derivatives as high-spin or spin-switchable materials. We are presently exploring strategies for increasing the diradical character of 2.

## Author contributions

BKH completed synthesis; DMC and MPN collected time-resolved spectroscopic data; BKH, DMC and TWS analysed the spectroscopic data; SSC collected crystallographic data; BKH, SDSB, KMP, RGP, TWS and MDP conducted computational chemistry calculations; BKH, RGP and MDP drafted the manuscript and all authors reviewed and edited it.

## Conflicts of interest

There are no conflicts to declare.

## Supplementary Material

SC-016-D5SC01676B-s001

SC-016-D5SC01676B-s002

## Data Availability

The data supporting this article have been included as part of the ESI.† Cartesian coordinates have been deposited on Figshare: https://doi.org/10.6084/m9.figshare.28827260. Crystallographic data for 10 have been deposited at the CCSD under 2427684.
