# Supplementary material for: Photoexcited and ground-state diradical(oid) character in a triquino[3]radialene
Source: Chem Sci. 2025 May 20;16(25):11331–8. doi: 10.1039/d5sc01676b (PMC12124304; doi:10.1039/d5sc01676b)
Supplement: SC-016-D5SC01676B-s001 [file SC-016-D5SC01676B-s001.pdf]

Supplementary Information (SI) for Chemical Science.
This journal is © The Royal Society of Chemistry 2025

Electronic Supplementary Information

for

Photoexcited and ground-state diradical(oid) character in a triquino[3]radialene

Bethany K. Hillier,[a] Damon M. de Clercq,[a] Stephen D. S. Bortolussi,[a] Simona S. Capomolla,[a] Michael P. Nielsen,[b] Katarzyna Młodzikowska-Pieńko,[c] Renana Gershoni-Poranne,[c] Timothy W. Schmidt,[a] Martin D. Peeks[a,*]

a. School of Chemistry, UNSW Sydney, NSW 2052 Australia
b. School of Photovoltaics and Renewable Energy Engineering, UNSW Sydney, NSW 2052 Australia
c. Schulich Faculty of Chemistry, Technion-Israel Institute of Technology, Technion City, Haifa 32000, Israel

Contents

1. Synthetic procedures S2
2. Spectroscopic details and supplementary data S5
  2.1. Instrumentation S5
  2.2. Fitting of Time-Resolved Spectroscopy Data S5
3. Computational details and supplementary data S14
  3.1 DFT calculations S14
  3.2 CIPT2 calculations S14
  3.3 Quantitative indicators of aromaticity S16
  3.4 Magnetically-induced currents S17
  3.5 Anisotropy of the induced current density (ACID) S21
  3.6 NRT analysis and NBO spin populations S25
4 Additional supplementary data S26
  4.1. Electronic spectra S26
  4.2. Vibrational spectra S30
  4.3. NMR spectra S32
  4.4. X-ray crystallography S37
5 References S38

1. Synthetic procedures

Solvents and reagents were purchased from Sigma-Aldrich (Merck), Combi-Blocks, Chem-Supply, and Fluorochem Limited and used without further purification. CH_2Cl_2 was dried using an MBraun solvent purification system. Reactions were performed under an inert atmosphere of N_2 using standard Schlenk techniques.

NMR spectroscopy was performed at room temperature using a 400 MHz Bruker Avance III HD spectrometer fitted with a Prodigy nitrogen-cooled cryoprobe. NMR samples were prepared using $CDCl_3$ purchased from Novachem, stored over K_2CO_3, and filtered through alumina immediately before use. Signals in proton NMR spectra are described as follows: singlet (s), multiplet (m), and broad (br). NMR spectra were processed with Bruker TopSpin 4.0.7.

Mass spectrometry was conducted using a Bruker ultrafleXtreme MALDI-ToF/ToF with trans-2-[3-(4-tert-Butylphenyl)-2-methyl-2-propenylidene]malononitrile (DCTB) as a matrix.

UV-vis spectra were measured on a Cary 60 single-beam UV/Vis spectrophotometer, using a quartz cuvette of pathlength 1 cm. Absorbance in the NIR region was measured using a PerkinElmer Lambda 1050 UV/Vis/NIR spectrophotometer using a quartz cuvette of pathlength 1 cm.

Infrared spectroscopy was performed on a solid sample using a ThermoFisher Nicolet iS50 FTIR spectrophotometer equipped with an ATR accessory, and processed with OMNIC software.

Prior to analysis by transient absorption spectroscopy, purity of samples was verified by LC-UV-MS on a ThermoScientific QExactive HF instrument, using a C8 column, eluting with 100% acetonitrile. The retention time of the radialene **2** was 23.4 minutes (Figure S40). No other peaks were observed in the sample.

Synthesis of diarylquinocyclopropene ***10***

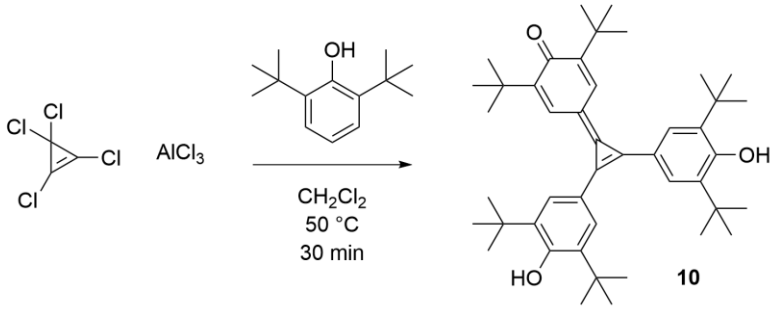

Scheme S1: Synthesis of diarylquinocyclopropene **10.**

Tetrachlorocyclopropene (0.2 mL, 1.63 mmol) was added to $AlCl_3$ (0.22 g, 1.65 mmol), and heated for 3 minutes at 80 °C to promote formation of trichlorocyclopropenium

tetrachloroaluminate. A solution of 2,6-di-*t*-butylphenol (1.12 g, 5.43 mmol) in dry CH_2Cl_2 (2 mL) was added dropwise, and the reaction mixture stirred at 50 °C for 30 minutes. The reaction was quenched with water (10 mL) to produce an oily orange residue. The aqueous layer was separated and the organic material was extracted with CH_2Cl_2 (5×20 mL). The combined organic extracts were washed with brine, dried over magnesium sulfate, and stripped of solvent under reduced pressure to afford the crude product. Layer recrystallisation (CH_2Cl_2/hexane) and trituration with acetonitrile gave **10** as a bright orange/red powder (0.802 g, 1.23 mmol, 75%).

The same reaction conditions are reported to also afford a charged species, tris(3,5-di-t-butyl-4-hydroxyphenyl)cyclopropenium chloride, which can be treated with base to give the neutral species **10**.[1] The charged species can be regenerated from **10** with the addition of acid.[1]

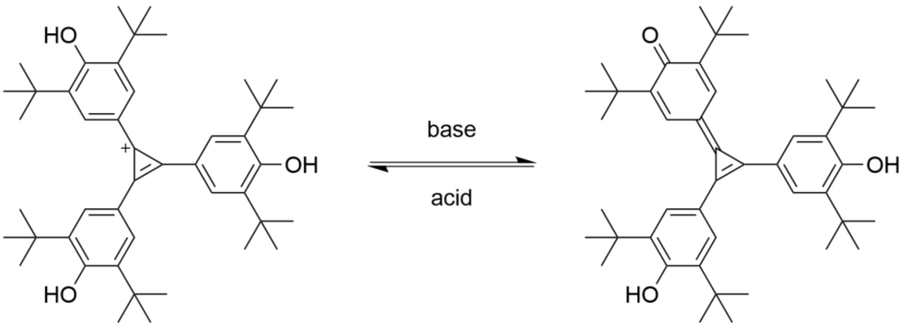

tris(3,5-di-t-butyl-4-hydroxyphenyl)cyclopropenium chloride

10

Scheme S2: Acid/base mediated conversion between cyclopropenium salt (left) and diarylquinocyclopropene **10** (right).

Though no base was added to the reaction, the species we isolated proved to be the neutral diarylquinocyclopropene **10**. The structure was confirmed by X-ray crystallography (Figure S41) and the electronic spectrum is in good agreement with literature values reported for the neutral compound.[1] Finally, with the addition of acid to **10** in solution, the electronic spectrum closely matches that reported for the charged cyclopropenium species (Figures S25-27, Table S8).[2]

^{1}H NMR ($CDCl_3$, 400 MHz): δ ppm 7.92 (s, 1H, aromatic), 5.97 (s, br, OH), 1.53 (s, 9H, t-butyl).

^{13}C NMR ($CDCl_3$, 100 MHz): δ ppm 166.3, 139.2, 138.9, 130.3, 109.3, 34.7, 30.1.

UV-vis λ_{max} nm (CH_2Cl_2) (log ε M^{-1} cm^{-1}): 310 (4.402), 326 (4.583), 385 (4.575), 410 (4.879).

Mass spec. (MALDI-ToF): *m/z* = 651.71 (calc. for [M]+ = 651.5)

*Synthesis of triquino[3]radialene **2***

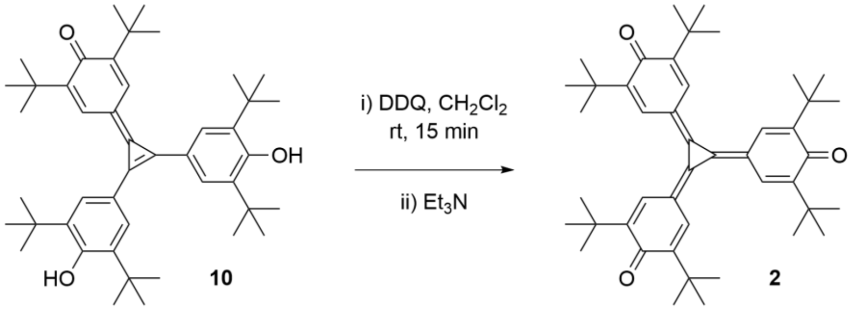

Scheme S3: Synthesis of triquino[3]radialene **2**.

DDQ (0.109 mg, 0.482 mmol) was added to a solution of **diarylquinocyclopropene 10** (0.152 g, 0.233 mmol) in dry CH_2Cl_2 (2 mL) and immediately caused a color change from orange to blue/black. The reaction mixture was stirred at room temperature for 15 minutes, then quenched with Et_3N (1 mL). Excess DDQ was removed by passage of the mixture through a silica plug eluting with CH_2Cl_2. Layer recrystallisation (CH_2Cl_2/hexane) of the concentrated eluent and trituration with acetone afforded **2**, the triquino[3]radialene, as a dark blue powder (0.097 g, 0.149 mmol, 64%).

^{1}H NMR ($CDCl_3$, 400 MHz) δ ppm 7.57 (s, 1H, aromatic), 1.40 (singlet, 9H, *t*-butyl).

^{13}C NMR ($CDCl_3$, 100 MHz) δ ppm 186.0, 150.8, 131.0, 130.3, 120.9, 36.0, 30.0.

UV-vis λ_{max} nm (log ε M^{-1} cm^{-1}): 310 (4.111), 389 (4.514), 629 (4.303), 686 (4.532), 767 (4.524).

Mass spec. (MALDI-ToF): *m/z* = 648.69 (calc. for [M]+ = 648.45).

The above oxidation procedure, substituting dichloromethane for trifluorotoluene or acetonitrile, gave **2** in 49% and 42% yields respectively. **2** was alternatively prepared in 28% yield by oxidation of the diarylquinocyclopropene **10** with 1.5 equivalents of chloranil according to the above procedure. Oxidation of **10** with 3 equivalents of aqueous basic $K_3[Fe(CN)_6]$ and subsequent aqueous workup, extracting organic material with CH_2Cl_2, also gave **2** in 45% yield. The latter experiment was also performed using trifluorotoluene as organic solvent, yielding 20% of **2**. These reaction outcomes are presented in Table S1.

Table S1: Synthetic yields of **2**, prepared in different organic solvents and with different oxidants.

Solvent	Oxidant	Yield
Trifluorotoluene	$K_3[Fe(CN)_6]$	20%
Trifluorotoluene	2,3-dichloro-5,6-dicyano-1,4-benzoquinone (DDQ)	46%
Acetonitrile	DDQ	40%
CH_2Cl_2	$K_3[Fe(CN)_6]$	43%
CH_2Cl_2	Chloranil	28%
CH_2Cl_2	DDQ	64%

2. Spectroscopic details and supplementary data

2.1. Instrumentation

Transient absorption (TA) measurements were performed on a commercial instrument (Ultrafast Systems, Helios) pumped by a Ti:Sapphire amplifier (Spectra Physics Solstice Ace) that generates an 800 nm pulse train at a repetition rate of 1 kHz with a pulse duration of nominally 100 fs. The 800 nm output was split with a beam splitter and one part of the pulse was sent to an optical parametric amplifier (TOPAS Prime, Light Conversion) to generate the pump pulse at 800 nm. A 550 nm long-pass filter was placed in the pump line to block residual 532 nm light. Before the sample, the pump polarization was set to the magic angle (54.7°). The second part of the 800 nm pulse was focused onto a nonlinear crystal to generate the white light continuum probe. An 8 ns optical delay line with a minimum step size of 14 fs varied the pump-probe delay. The signal was detected with a silicon linear array detector for the UV-vis region and an InGaAs linear array detector for the NIR region. At least three individual scans were taken, and each scan was averaged over 3 s per time delay. For the long-time TA, an ND:YVO_4 laser system (Piccolo Innolas, 355 nm, 800 ps pulse duration) set to external frequency (500 Hz) control was used as the pump beam. The pump-probe delay was controlled electronically via a delay generator triggering the pump pulse.

2.2. Fitting of Time-Resolved Spectroscopy Data

2.2.1 Sequential model

The TA data was first chirp corrected, then global lifetime analysis was performed using python modelling package KiMoPack.[3]

Modelling the visible and NIR regions independently returned similar rates and lifetimes as when the regions were modelled together. We therefore followed the latter approach, and modelled both the visible and NIR regions together using a consecutive model. In all cases, the dataset was best described by a two-states model, assigned as S_1 and T_1 (see main text). Including a third species in the fit marginally improved the R^2 values, however had no impact on the lifetimes of S_1 and T_1. Further, the spectrum associated with the third species was a superposition of the other two species' spectra.

The time region between –6 and 0.5 ps was removed from the optimization, and absorbance between 765 and 815 nm removed due to scatter from the pump laser. Rates and lifetimes were calculated with 95% confidence intervals. The lifetime of T_1 is not captured by this fitting model, as T_1 persists for longer than the experiment.

The lifetimes of S_1 and T_1 in different solvents, and associated R^2 values, are tabulated in Table S2. Spectra of ΔA vs time for S_1 and T_1 are presented in Figure S1 and Figure S2, respectively.

Table S2: Results of fitting TA data of **2** in different solvent compositions to a sequential model; solutions of **2** had OD of 0.3 (toluene 0.1 OD), pump wavelength 800 nm, delay line up to 7 ns.

**Solvent**	**S_1 lifetime (ps)**	**95% confidence interval for S_1 lifetime (ps)**	**T_1 lifetime (ns)**	**R^2**
**CH_2Cl_2**	9.06	8.64–9.51	n.d.[a]	0.988
**Toluene**	10.40	8.11–13.42	n.d.[a]	0.677
**2% MeOH in CH_2Cl_2**	8.67	7.35–10.27	n.d.[a]	0.785
**5% MeOH in CH_2Cl_2**	8.18	6.58–10.21	n.d.[a]	0.610
**10% MeOH in CH_2Cl_2**	7.95	6.94–9.31	n.d.[a]	0.797
**20% MeOH in CH_2Cl_2**	7.77	5.46–11.1	n.d.[a]	0.403

[a] The T_1 state has a lifetime longer than the timescale of this experiment (see below). The fitting algorithm returned spurious lower-bounds on the lifetime of between 20 and 8×10^8 ns.

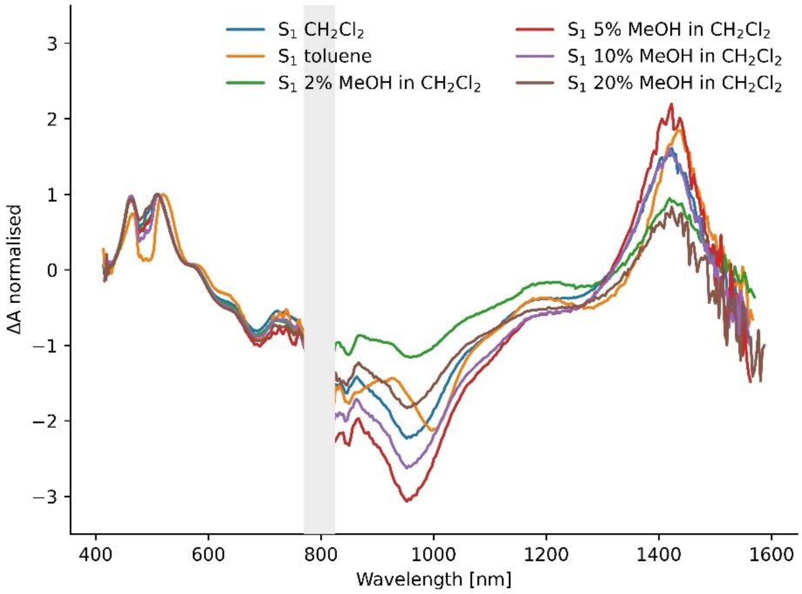

Figure S1: Spectra of S_1 after excitation at 800 nm, normalized to maximum of peak between 507 and 519 nm.

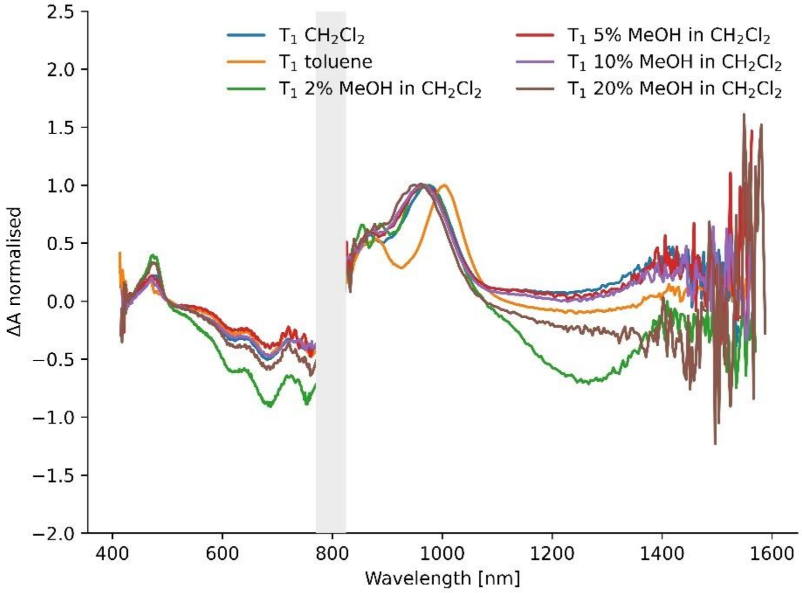

Figure S2: Spectra of T_1 after excitation at 800 nm, normalized to maximum of peak between 949 and 1004 nm.

2.2.2 Monoexponential fitting model

The transient absorption data for **2** in CH_2Cl_2, toluene, and 2-20% MeOH in CH_2Cl_2 were fit with a monoexponential of general equation:

$$y = Ae^{-kt}$$

from which the lifetime, τ, of the S_1 state was calculated as:

$$\tau = \frac{1}{k}$$

The signal at 510 nm was chosen to calculate the lifetime of S_1 as there is relatively little contamination from T_1, which does not absorb in this region (Figure 2(a), SI Figure S9).

The lifetime of S_1 in different solvents calculated from the monoexponential model are presented in Table S3, and plots of ΔA vs time for each solvent in Figures S3-S8.

Table S3: Results of fitting the TA data to a monoexponential model for S_1

**Solvent**	**Lifetime of S_1 (ps)**	R^2
**CH_2Cl_2**	7.00	0.994
**Toluene**	8.65	0.989
**2% MeOH in CH_2Cl_2**	7.03	0.994
**5% MeOH in CH_2Cl_2**	6.92	0.993
**10% MeOH in CH_2Cl_2**	6.78	0.994
**20% MeOH in CH_2Cl_2**	6.49	0.994

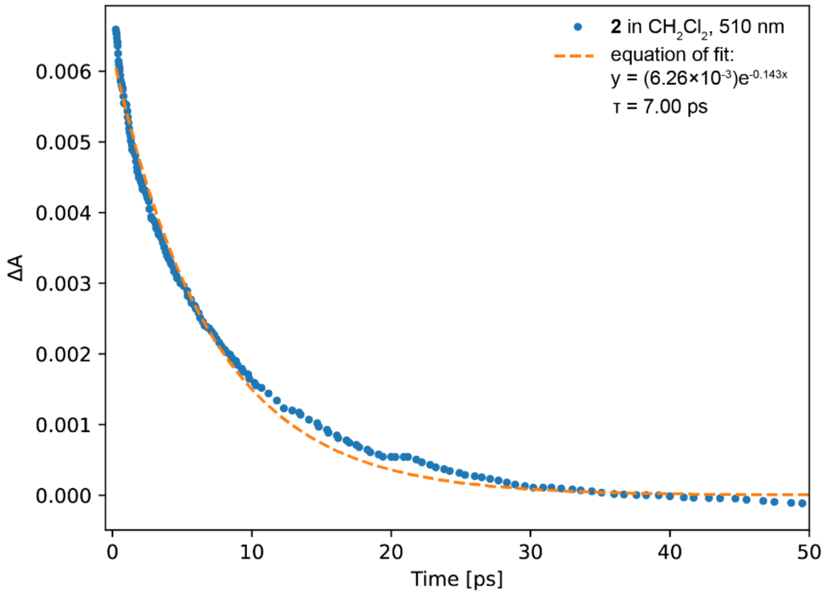

Figure S3: ΔA_{510} *vs*. time for **2** in CH_2Cl_2 after excitation at 800 nm (dots), fit to a monoexponential (dashed line).

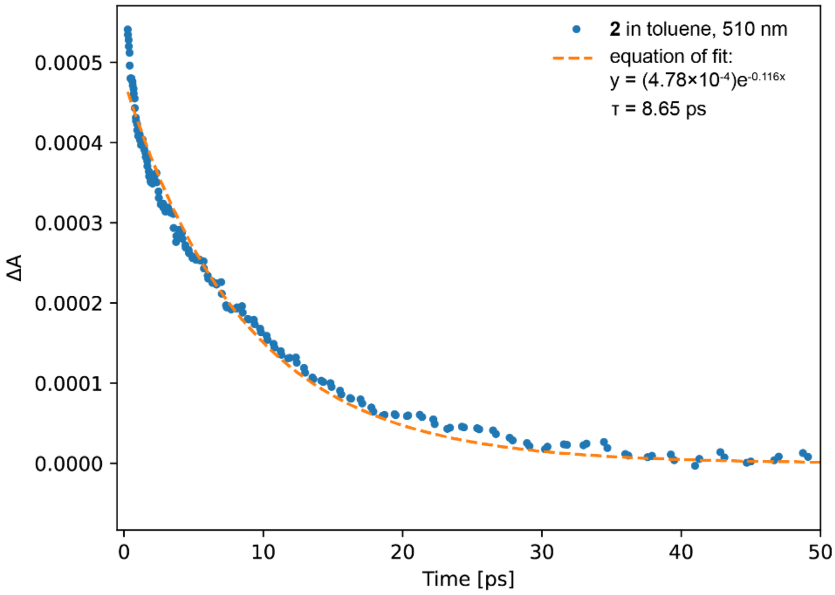

Figure S4: ΔA_{510} *vs*. time for **2** in toluene after excitation at 800 nm (dots), fit with a monoexponential (dashed line).

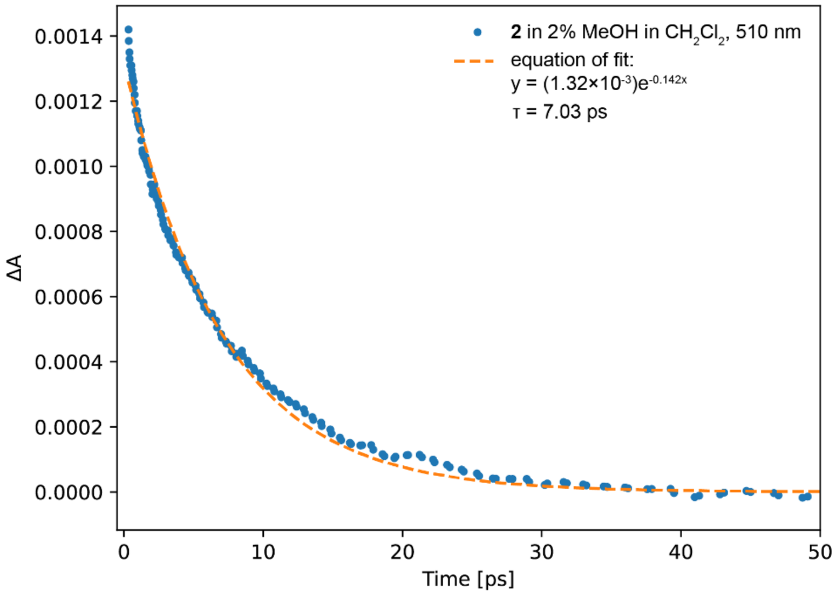

Figure S5: ΔA_{510} *vs*. time for **2** in 2% MeOH/CH_2Cl_2 after excitation at 800 nm (dots), fit to a monoexponential (dashed line).

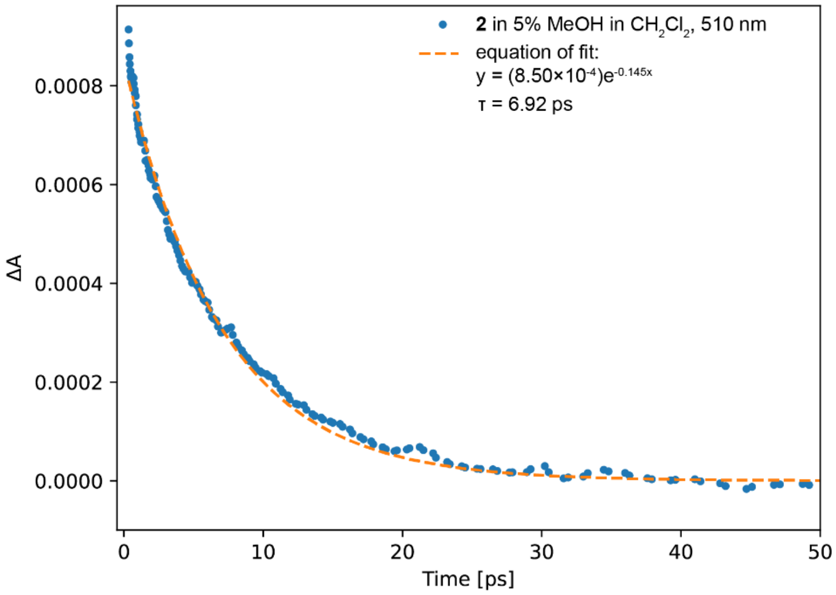

Figure S6: ΔA_{510} *vs*. time for **2** in 5% MeOH/CH_2Cl_2 after excitation at 800 nm (dots), fit to a monoexponential (dashed line).

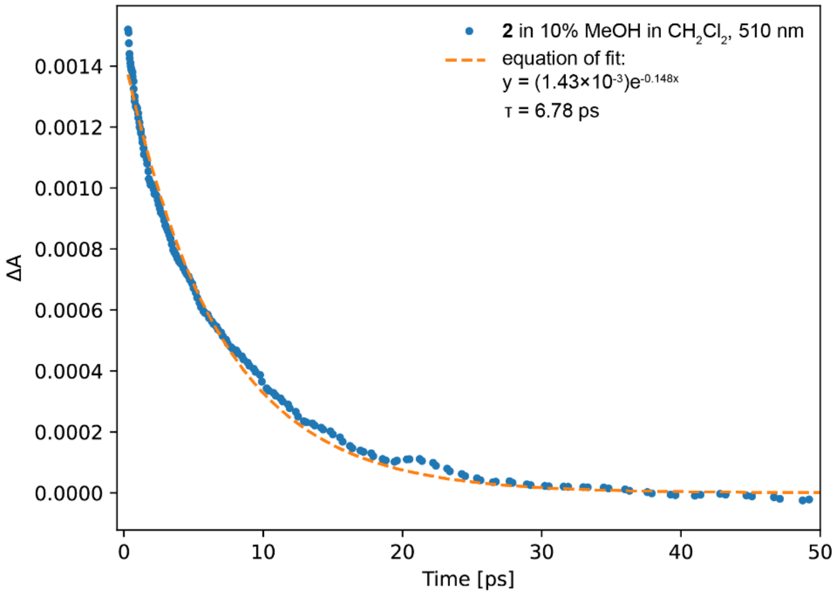

Figure S7: ΔA_{510} *vs*. time for **2** in 10% MeOH/CH_2Cl_2 after excitation at 800 nm (dots), fit to a monoexponential (dashed line).

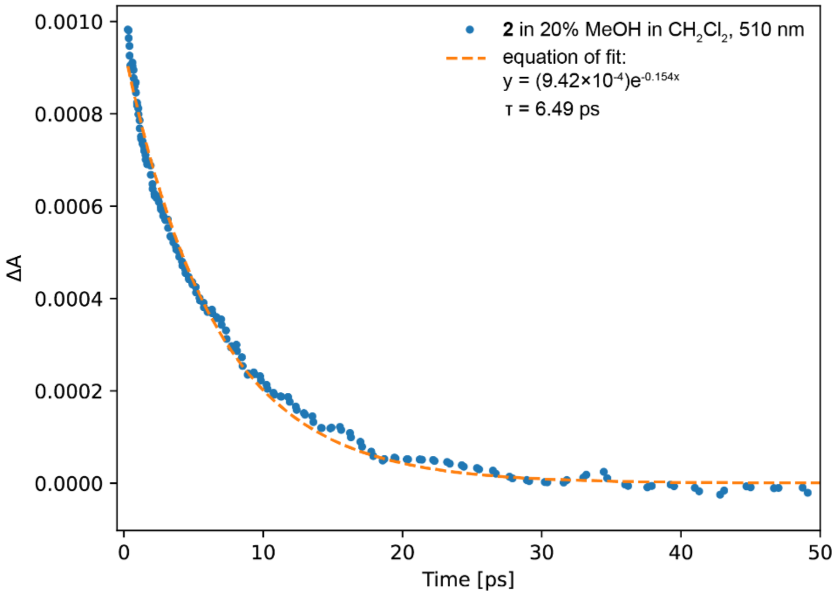

Figure S8: ΔA_{510} *vs*. time for **2** in 20% MeOH/CH_2Cl_2 after excitation at 800 nm, fit to a monoexponential (dashed line).

2.2.2.1 Lifetime of T_1: long-time TA experiment

Transient absorption between 409 and 780 nm of **2** in CH_2Cl_2 was measured on a microsecond timescale, to determine the lifetime and spectrum of T_1.

Using KiMoPack, the data between 450 and 750 nm were fit to a sequential model. The time region -0.1 and 0.2 μs was removed from the fit. Since 0.2 μs is longer than the lifetime of S_1, the data were fit only using one species. When a second species was added, its spectrum overlapped with that calculated for T_1. Using this fit model, the lifetime of T_1 was determined as 0.82 μs (R^2 = 0.945).

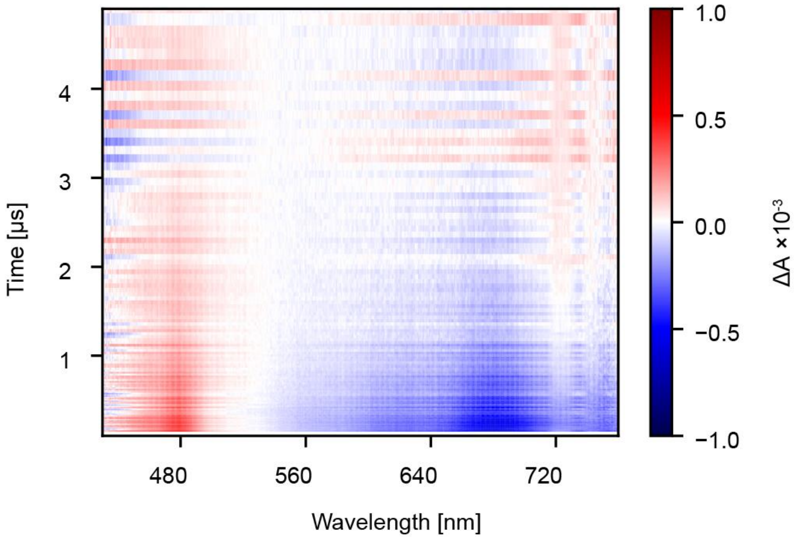

Figure S9: Transient absorption of **2** in CH_2Cl_2 after excitation at 355 nm, microsecond timescale.

The long-time TA data were also fit using a monoexponential model. When the bleach at 681 nm was fit using this method, the lifetime of T_1 was found to be 0.92 μs (Figure S10). The lifetime measured from fitting the excited-state absorption at 474 nm was 1.2 μs (Figure S11).

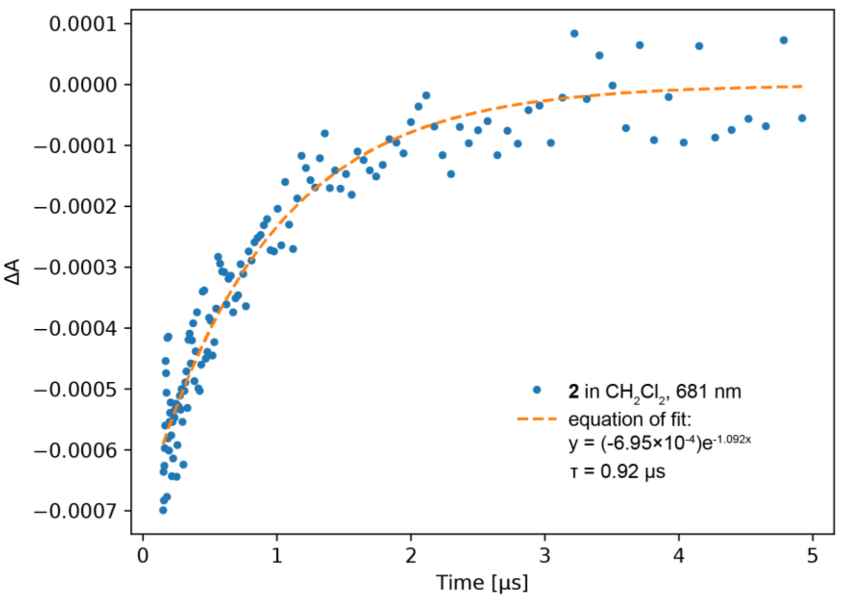

Figure S10: ΔA_{681} *vs.* time of **2** in CH_2Cl_2 after excitation at 355 nm (Nd:YAG laser) (dots). Fit to a monoexponential decay (dashed line).

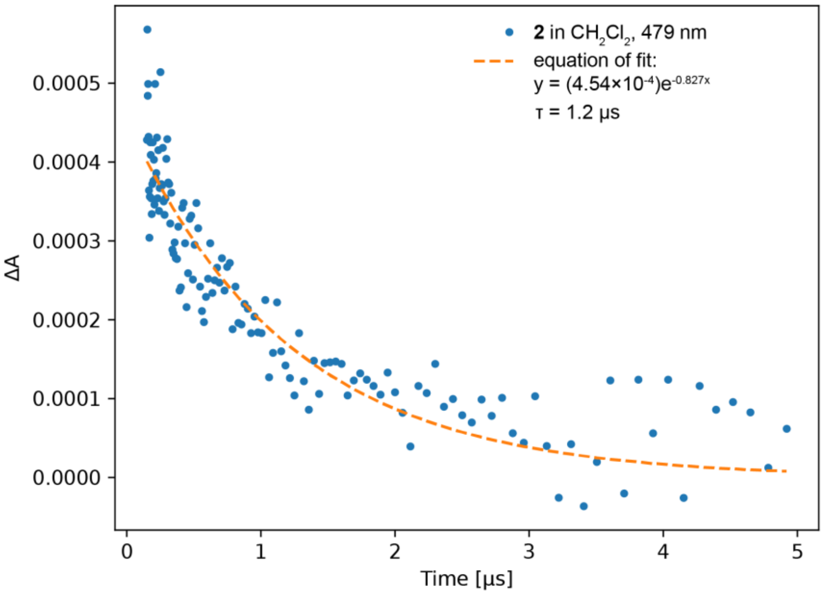

Figure S11: ΔA_{479} vs time of **2** in CH_2Cl_2 after excitation with 355 nm (Nd:YAG laser) (dots). Fit to a monoexponential decay (dashed line).

3. Computational details and supplementary data

3.1 DFT calculations

Initial geometries were pre-optimized with XTB[4] and then further optimized with Gaussian16/A.03 at the CAM-B3LYP/6-31G* level using default convergence criteria.[5–9] Geometries were confirmed as minima by the absence of imaginary (negative) frequencies in frequency calculations. TD-DFT calculations used both CAM-B3LYP and B3LYP[10,11]; B3LYP provided a better match to the experimental absorption spectrum.

3.2 CIPT2 calculations

To allow the wavefunction calculations to exploit symmetry, **2** and Yang's biradical were re-constrained to planarity, with tert-butyl groups truncated to –H, and re-optimized using CAM-B3LYP/6-31G* in Gaussian16/A.03. For **2**, the resulting structure was a first-order transition state with $\nu = -12\ \text{cm}^{-1}$, suggesting that the planar structure is thermally accessible to the bare π-system. Excited state calculations were performed using the Molpro 2020.1.[12–14] A restricted Hartree-Fock (RHF) calculation was performed with the cc-pVDZ basis,[15] yielding orbitals that respected the D_{3h} geometry provided. In C_{2v} symmetry, the A_1 and B_2 representations span the s-space, and the B_1 and A_2 representations span the p-space. The closed-shell RHF wave function occupied $38A_1$, $7B_1$, $31B_2$ and $5A_2$ orbitals (162 electrons). The energies of the π-orbitals are listed below with their symmetry labels given in the D_{3h} point group. The occupied orbitals are given in bold.

Label (D_{3h})	B_1	
E"	+0.062105	LUMO+1
A_2"	-0.073991	LUMO
**E"**	**-0.312591**	**HOMO**
**A_1"**	**-0.402945**	**HOMO-1**
**E"**	**-0.404381**	
**A_2"**	**-0.431443**	
**E"**	**-0.493805**	
**A_2"**	**-0.556068**	
**E"**	**-0.573974**	
**A_2"**	**-0.606253**	

An MCSCF calculation was performed on the ground state, including the orbitals listed in the table, excluding those shaded. The leading coefficient in terms of the natural orbitals was found to be the closed shell configuration, with a magnitude of 0.934. The next most important configurations are double-excitations from each of the E" HOMOs into the A_2" LUMO (-0.145). As such, there is non-negligible diradical character in the ground state wave function. MCSCF locates a degenerate S_1 state mostly described by the HOMO-LUMO excitation. The transition moment is calculated to be 2.53 ea_0.

A CIPT2 calculation[16] was performed with a small active space comprising the HOMO, the LUMO and the LUMO+1. All excitations arising from the active space alone are treated by MRCI (all single and double excitations into external space). All remaining excitations are treated by second-order perturbation theory.

MCSCF was then performed, weighting the S_0 and degenerate S_1 state equally (0.33 each). The transition moment was calculated to be 2.93 ea_0 (f = 0.38). Using these orbitals, the S_0 and S_1 states were calculated by CIPT2 on an even footing. This predicts a 690 nm transition (695 nm with Davidson correction).

A minimal active space comprising the HOMO-1, HOMO(s) and LUMO also arrives at an excitation at 695 nm (CIPT2) with an oscillator strength of 4.15 ea_0.

The triplet states were calculated by weighting the degenerate components of the state equally. A strong T_2-T_1 transition is predicted at 879 nm with transition moment 1.92 ea_0 (f = 1.27).

Table S4: State energies from wavefunction calculations using Molpro (cc-pVDZ basis).

State	Method	Energy/E_h
S_0 A_1'	RHF	-1026.728853
	MCSCF (S_0 weighted)	-1026.802187
	CIPT2 (S_0 orbitals)	-1029.954668
	MCSCF (S_0/S_1 weighted)	-1026.793534
	CIPT2 (S_0/S_1 orbitals)	**-1029.968759**
		**-1029.968891 (Davidson)**
S_1 E'	MCSCF (S_0 weighted)	-1026.631754
	MCSCF (S_0/S_1 weighted)	-1026.673626
	CIPT2 (S_0/S_1 orbitals)	**-1029.902729**
		**-1029.903358 (Davidson)**
T_1 E'	MCSCF (T_1 weighted)	**-1026.725953**
	CIPT2 (T_1 orbitals)	**-1029.930886**
		**-1029.931148 (Davidson)**
T_2 A_1'	CIPT2 (T_1 orbitals)	**-1029.879315**
		**-1029.880524 (Davidson)**

Table S5: Summary of state energies for **2**, relative to the energy of S_0. DFT methods used a closed-shell wavefunction for S_0.

State	CIPT2 with Davidson correction		TD-B3LYP		TD-CAM-B3LYP	
	eV (nm)	*f* from MCSCF	eV (nm)	*f*	eV (nm)	*f*
S_0	0 (–)	–	0 (–)	–	0 (–)	–
T_1	1.03 (1207)	n.d.	0.46 (2730)[b]	0	–0.56 (–2207)[b]	0
S_1	1.78 (695)	0.38	1.67 (741)	0	2.33 (541)[b,c]	0.91
T_2	2.40 (516)	n.d.	1.44 (859)	0	2.17 (572)	-
S_2	3.12 (398)[a]	n.d.	1.72 (722)[b]	0.0001	2.50 (496)	0
S_3	n.d.	n.d.	1.98 (625)[b]	0.6565	2.56 (484)	0.001

[a] Calculated using [10,10] XMCQDPT2/cc-pVTZ
[b] Approximately doubly-degenerate in TD-DFT output.
[c] A broken-symmetry guess at the triplet geometry gives a state ($<S^2>$ after annihilation = 0.8364) which sits 0.1 eV below the optimized triplet state. A broken-symmetry guess at the singlet geometry gives a state (S^2 after annihilation = 0.0392) which sits only 0.01 below the closed-shell singlet.

3.3 Quantitative indicators of aromaticity

The FLU,[17] PDI,[18] I_{ring},[19] and MCI[20] values were calculated using the AIMAll,[21] and ESI-3D[22,23] packages using the CAM-B3LYP/6-31G* structure and wavefunction.

Table S6: Electronic metrics of aromaticity in triquino[3]radialene **2**, calculated using CAM-B3LYP/6-31G*.

	**Benzenoid ring 1**		**Quinoid ring**		**Benzenoid ring 2**	
	**Singlet**	**Triplet**	**Singlet**	**Triplet**	**Singlet**	**Triplet**
**FLU**	0.058919	0.031472	0.058919	0.050297	0.058919	0.031459
**PDI**	0.023021	0.045569	0.023019	0.028432	0.023020	0.045554
I_{ring}	0.007756	0.014362	0.007756	0.009762	0.007756	0.014359
**MCI**	0.008239	0.017151	0.008239	0.010728	0.008239	0.017149

Benzene reference values:[18,19,24]

PDI: 0.101

I_{ring}: 0.0883

MCI: 0.0484 (B3LYP/6-31G*),[20] 0.0716 (CAM-B3LYP/6-311+G(d,p))

Table S7: HOMA and HOMER values for the T_1 state of triquino[3]radialene and related molecules (cf. Table 1). Level of theory: CAM-B3LYP/6-31G*.

		**HOMA**	**HOMER**	**C=O bond length (Å)**
Galvinoxyl		0.092	–1.457	1.233
Yang's biradical **4**		0.249	–1.076	1.237
**2** T_1	Quinoid	−0.100	–1.906	1.236[a]
	Benzenoid	0.277	–1.010	1.245

[a] The C=O bond length in the singlet state of **2** is 1.229 Å.

3.4 Magnetically-induced currents

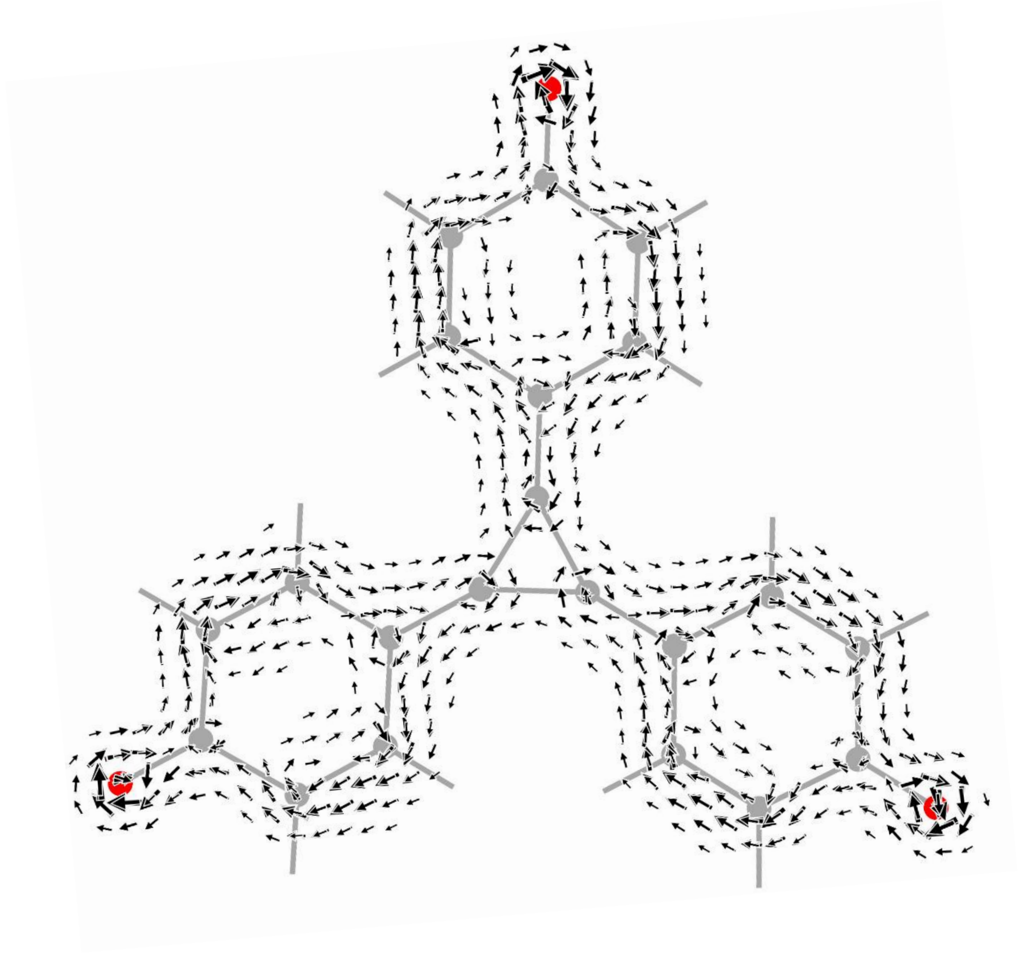

Figure S12: Magnetically-induced current density for π-electrons only, calculated using SYSMOIC, for a planarized model of **2** (tBu truncated to H), in its singlet (S_0) state. CAM-B3LYP/6-31G*. The current density was calculated 1.7 Å above the plane of the molecule.

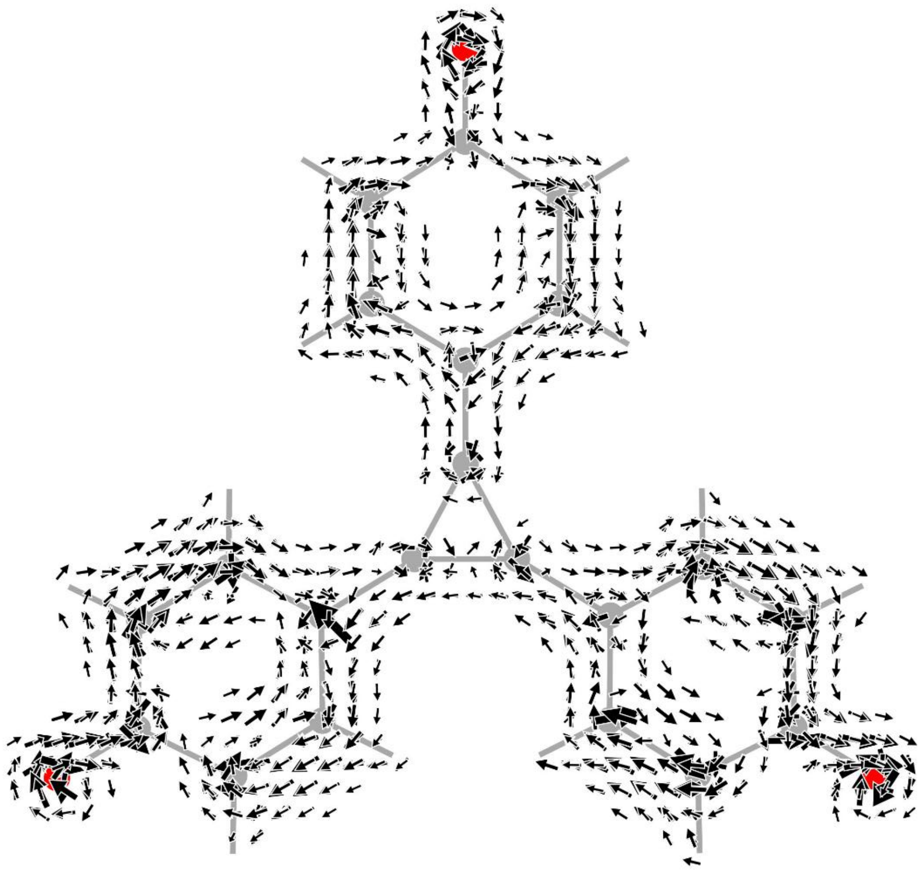

Figure S13: Magnetically-induced current density for π-electrons only, calculated using SYSMOIC, for a planarized model of **2** (tBu truncated to H), in its triplet (T_1) state. CAM-B3LYP/6-31G*. The current density was calculated 1.7 Å above the plane of the molecule.

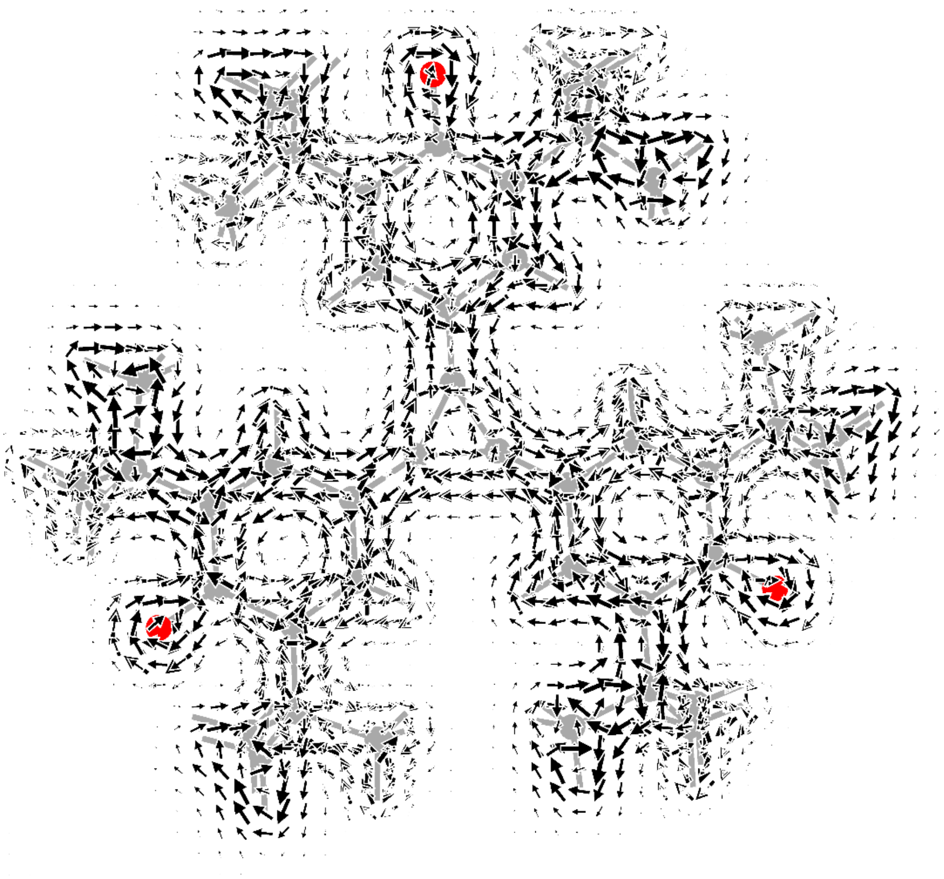

Figure S14: Magnetically-induced current density calculated using SYSMOIC for **2**, in its singlet (S_0) state. CAM-B3LYP/6-31G*. The current density was calculated 1.7 Å above the mean plane of the molecule.

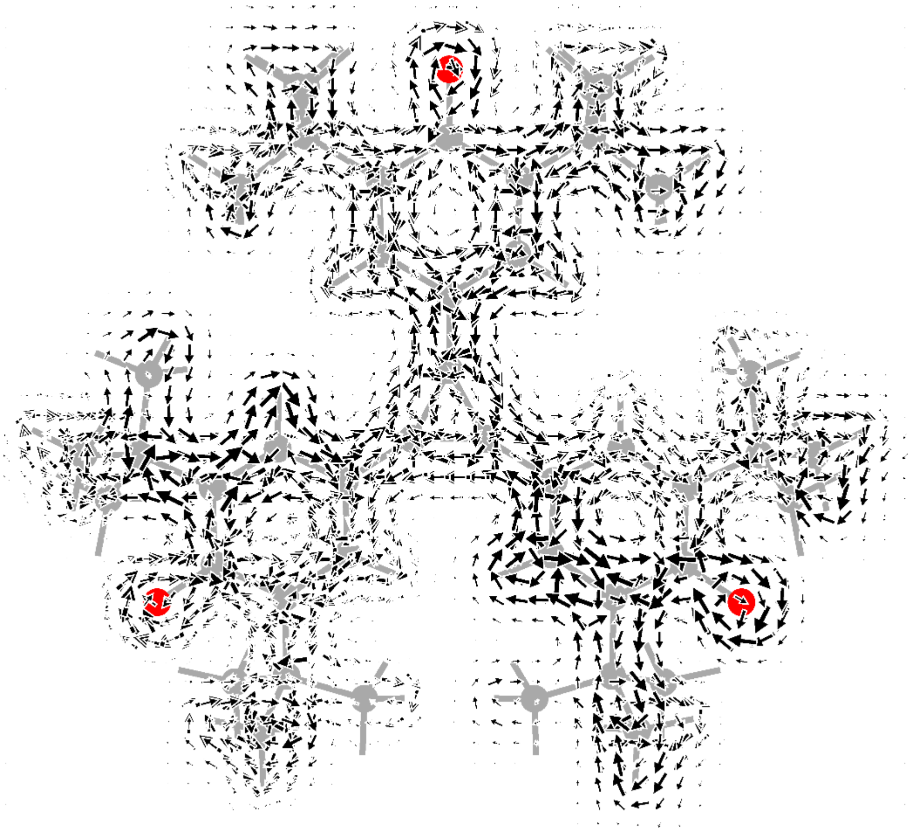

Figure S15: Magnetically-induced current density calculated using SYSMOIC for **2**, in its triplet (T_1) state. CAM-B3LYP/6-31G*. The current density was calculated 1.7 Å above the mean plane of the molecule.

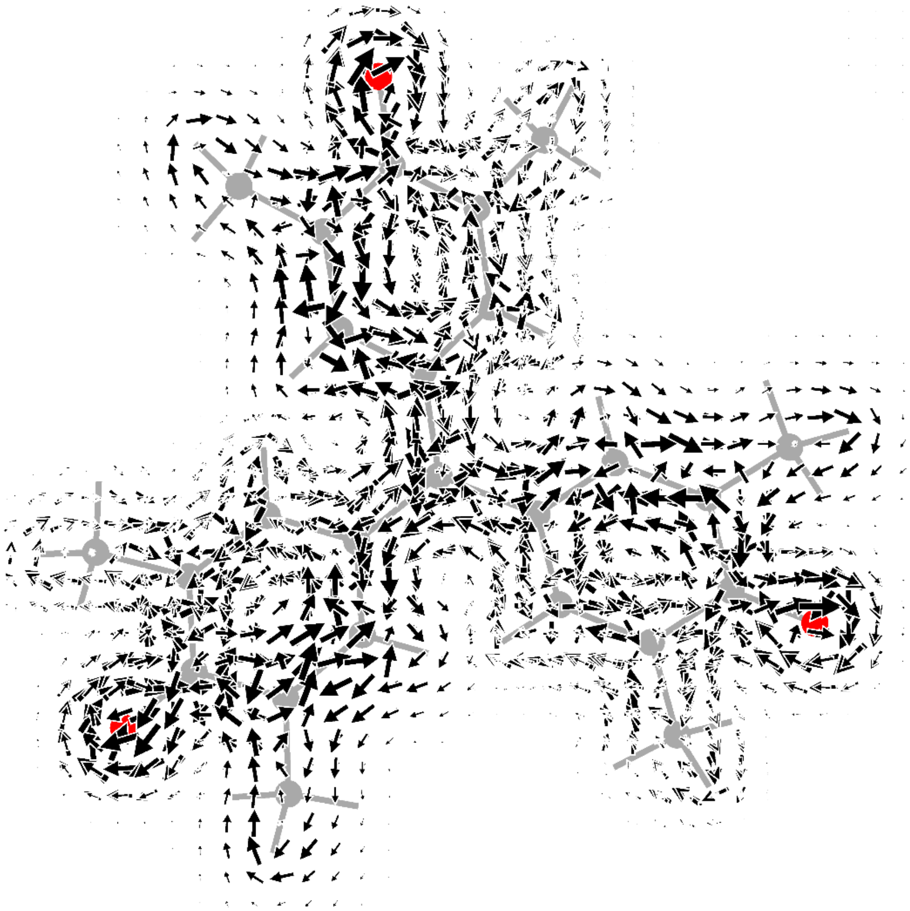

Figure S16: Magnetically-induced current density calculated using SYSMOIC for Yang's biradical **4**, in its triplet (T_1) state. CAM-B3LYP/6-31G*. The current density was calculated 1.7 Å above the mean plane of the molecule.

3.5 Anisotropy of the induced current density (ACID)

Analysis of the electronic delocalization of triquino[3]radialene **2** in its singlet (Figure S17) and triplet (Figure S18) states, and Yang's biradical **4** (Figure S19) was performed using the ACID method.[25,26] The vector arrows on the ACID isosurface show that there is no net current in each ring – rather the current density arrows flow around the whole molecule in each case.

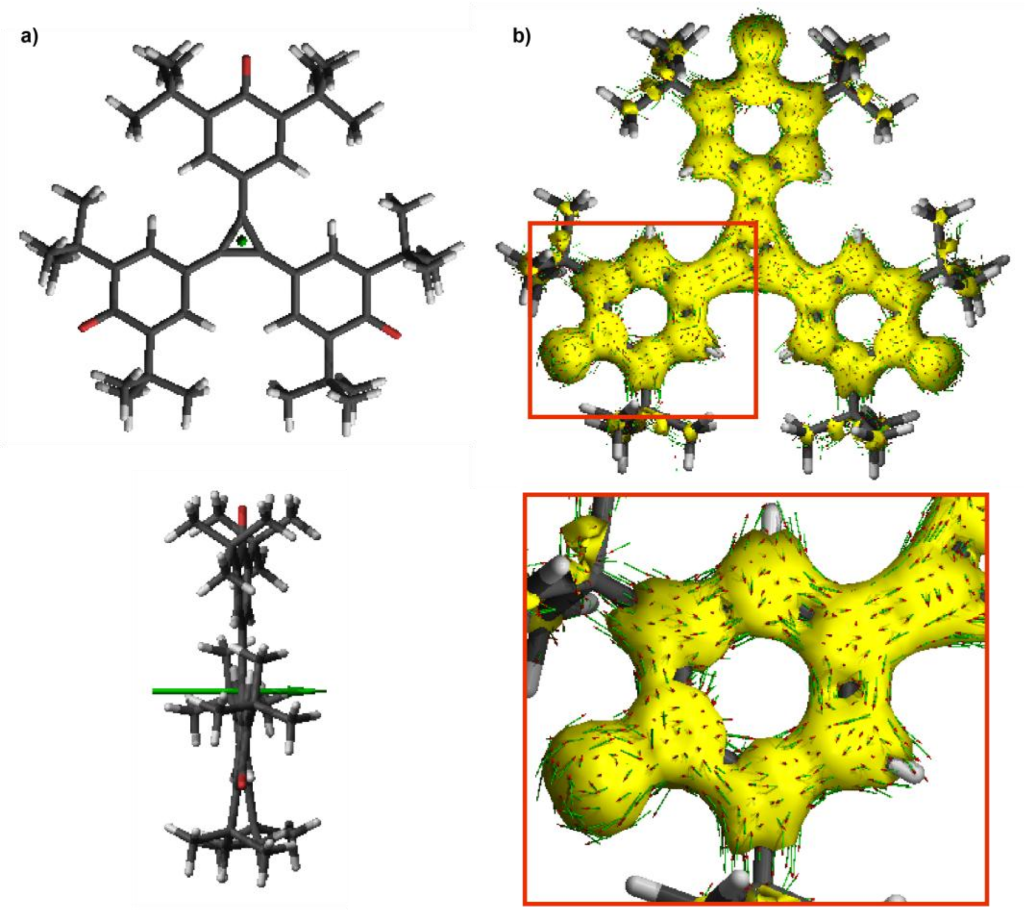

Figure S17: ACID of singlet **2**, geometry optimization and NMR calculation using CAM-B3LYP/6-31G*. Green arrow in **a)** shows direction of the external magnetic field. Isovalue = 0.050.

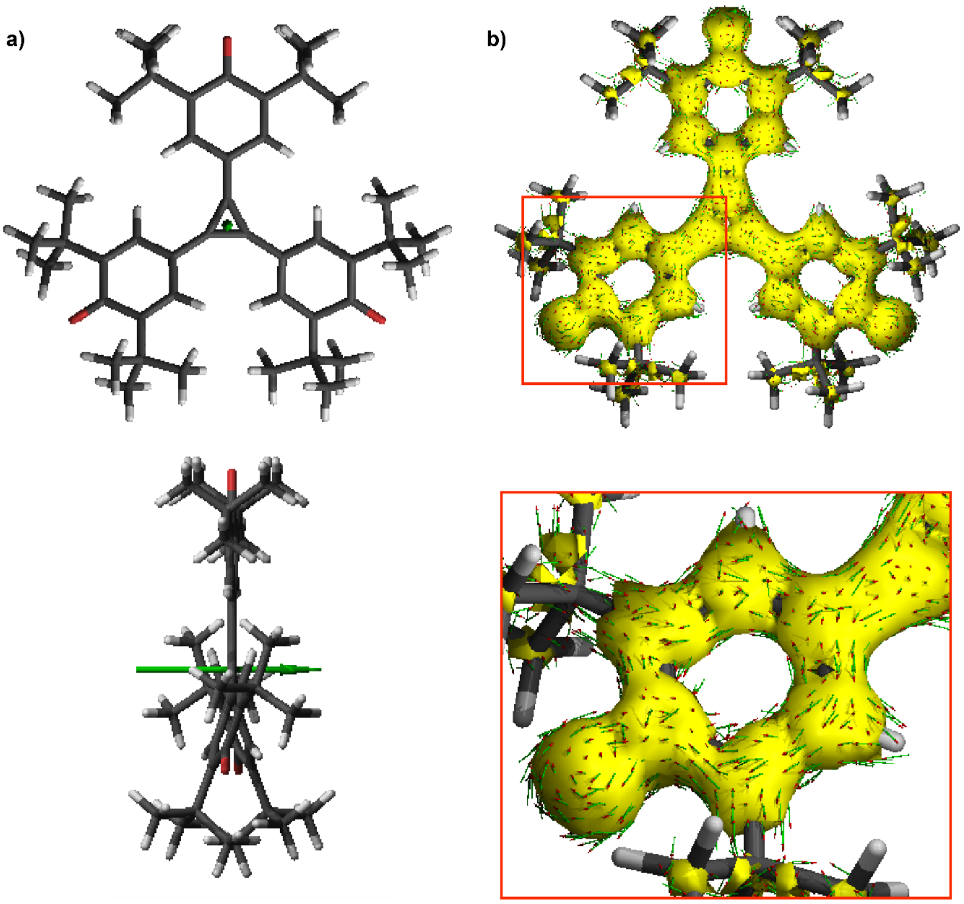

Figure S18: ACID of triplet **2**, geometry optimization and NMR calculation using CAM-B3LYP/6-31G*. Green arrow in **a)** shows direction of the external magnetic field. Isovalue = 0.050.

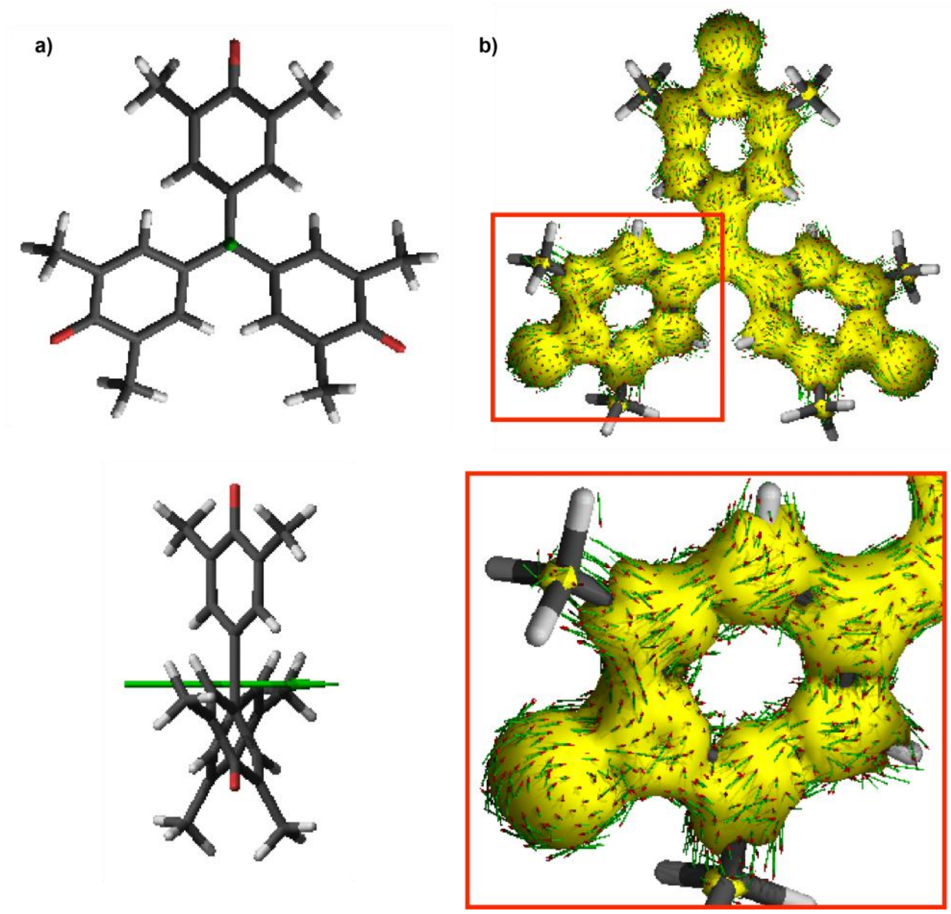

Figure S19: ACID of Yang's biradical **4** (*t*Bu groups truncated to Me), geometry optimization and NMR calculation using CAM-B3LYP/6-31G*. Green arrow in **a)** shows direction of the external magnetic field. Isovalue = 0.050.

3.6 NRT analysis and NBO spin populations

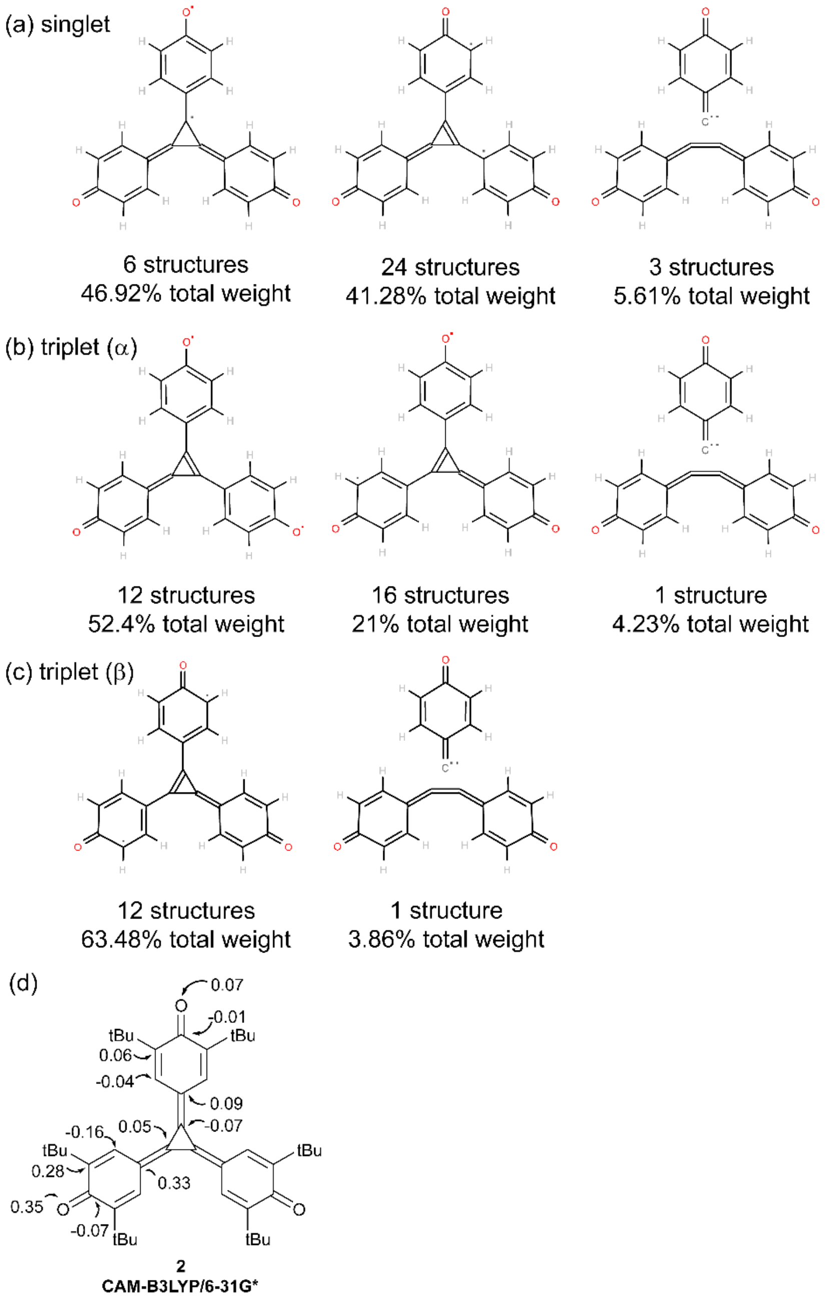

Figure S20: Natural resonance theory structures and natural spin population calculated using NBO 7.0.[27] (a) NRT structures for the singlet state of **2**; (b) NRT structures for the triplet state (alpha electrons) of **2**; (c) NRT structures for the triplet state (beta electrons) of **2**; (d) spin density (triplet state) from the NBO population analysis. In the NRT analyses, similar resonance structures have been grouped for presentation (i.e. “12 structures”); structures were grouped if the relative positions of radical centres were the same (e.g. one spin *ipso*, one *meta* on the C=C-linked ring). Strict symmetry was not followed. The NRT analysis used NRTE2=25%, and structures were extracted by converting the CML output (NRTCML) to an SVG using OpenBabel. In the NBO analysis, values are not provided for the *t*Bu groups; missing labels can be inferred by symmetry. Where spin populations differed between pseudo-symmetric atoms, an average (mean) value is presented; the resulting error is less than 0.01. Level of theory: CAM-B3LYP/6-31G*.

4 Additional supplementary data

4.1. Electronic spectra

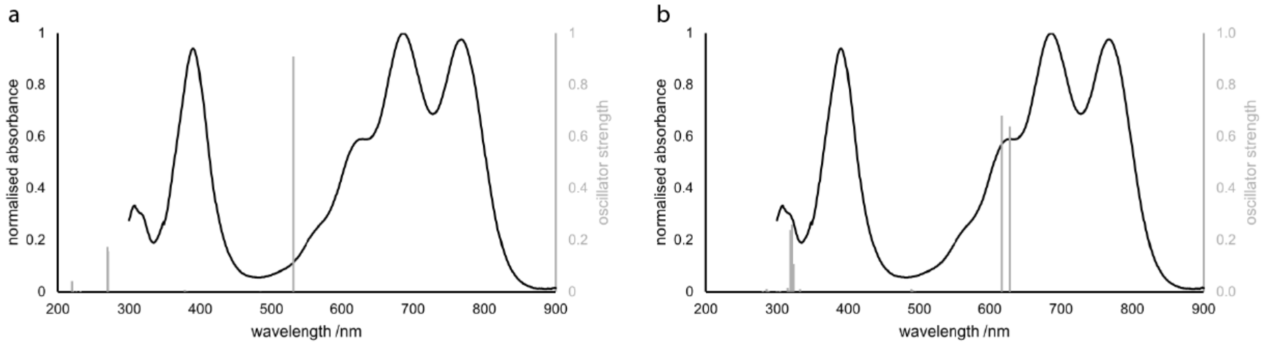

Figure S21: experimental UV-vis for singlet **2** (line) overlaid with calculated spectra (a) CAM-B3LYP/6-31G* (sticks), and (b) B3LYP/6-31G* (sticks)

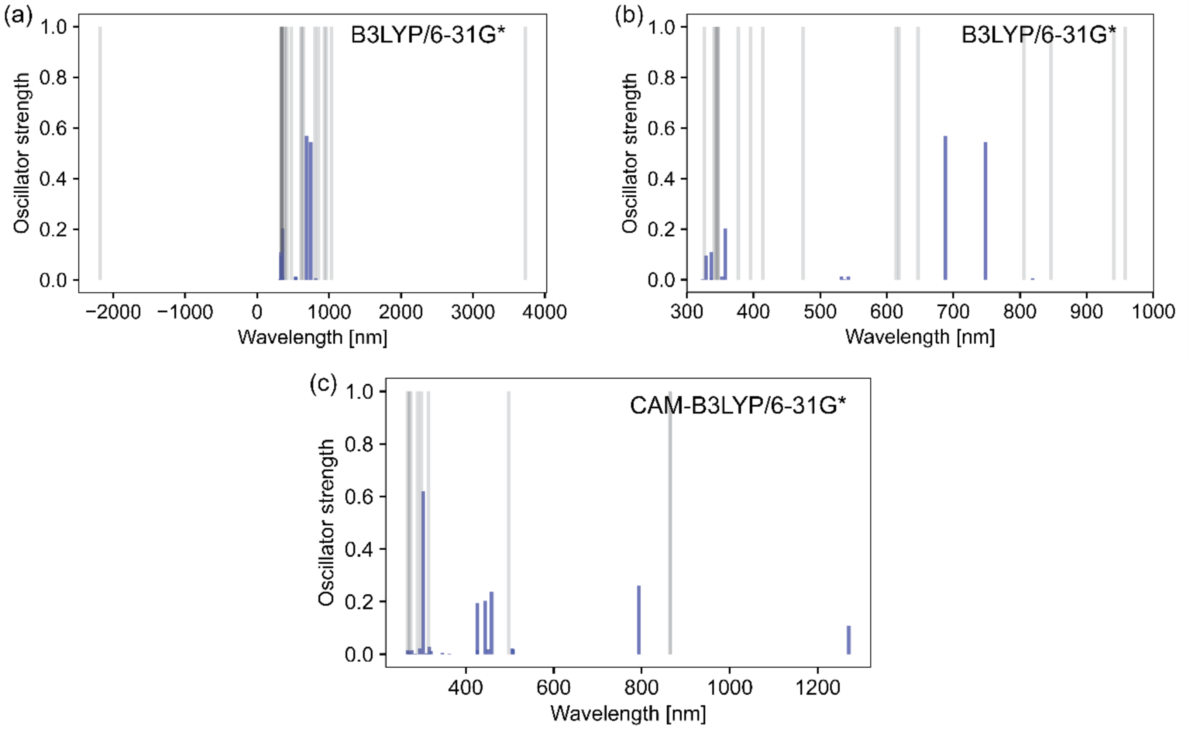

Figure S22: Calculated excitations from a singlet state of **2** generated using a broken-symmetry wavefunction guess on the triplet geometry. (a) B3LYP/6-31G*, which optimized to a singlet state (S^2=0) which was unstable with respect to a triplet state; (b) a cropped version of the spectrum; (c) CAM-B3LYP/6-31G*, which optimized to a stable singlet state (S^2 = 0.8364). Neither calculated spectrum offers a clear match to the experimental data. Forbidden transitions are shown as grey bars.

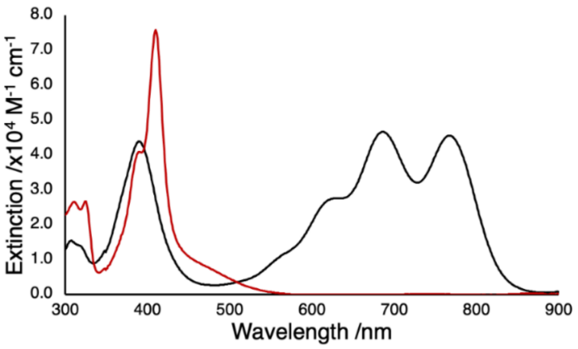

Figure S23: UV-vis spectra of diarylquinocyclopropene **10** and triquino[3]radialene **2** in CH_2Cl_2.

The electronic spectrum of the precursor **10** was also measured in acetonitrile and methanol. In all solvents, the spectrum is dominated by an absorption at ~410 nm. This is consistent with what is reported for the diarylquinocyclopropene in literature.[1]

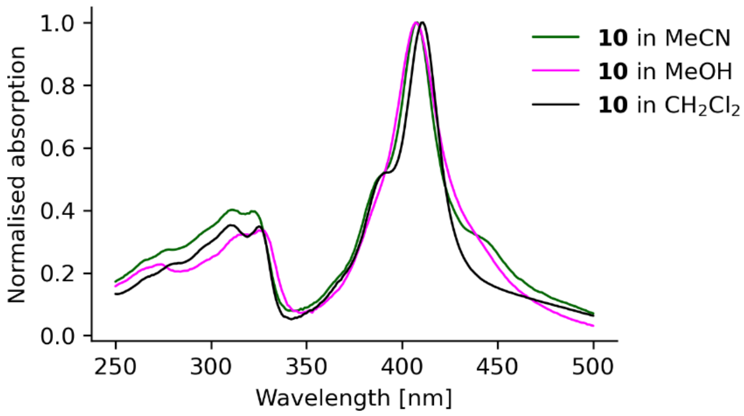

Figure S24: UV-vis spectra of diarylquinocyclopropene **10** in acetonitrile, methanol, and CH_2Cl_2. Normalized to λ_{max} in each spectrum.

In acetonitrile (Figure S25), the absorption maximum is at 408 nm, and there are discernible shoulders on either side of this peak at 390 and 440 nm. Higher energy transitions occur at 311 and 323 nm. There is an additional broad peak at 278 nm. When the solution is acidified by addition of HCl in dioxane, the solution turns from orange to colorless. The resulting spectrum closely resembles what is reported for the analogous cyclopropenium chloride in acetonitrile – with maximum absorption at 372 nm, and additional transitions at 242, 290, and 353 nm.[2] With the addition of base (Et_3N) to **10** in acetonitrile, the solution becomes yellow and there is a significant redshift in its absorption maximum from 408 to 442 nm, and there is a weaker transition at 320 nm. Similar changes were observed with the addition of acid and base to solutions of **10** in methanol (Figure S26) and CH_2Cl_2 (Figure S27). The spectrum of **10** in CH_2Cl_2 with base has fine structure that is not present for other solvents tested. The absorption maxima for each case are reported in Table S8.

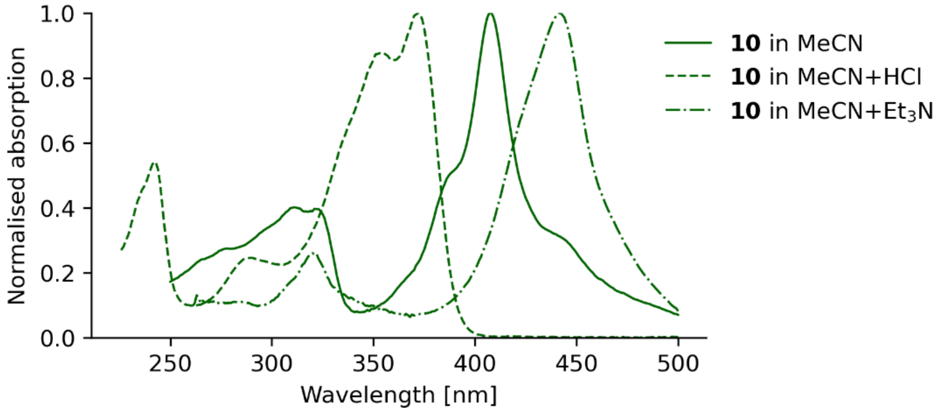

Figure S25: UV-vis spectra of diarylquinocyclopropene **10** in acetonitrile, and with the addition of acid and base. Normalized to λ_{max} in each spectrum.

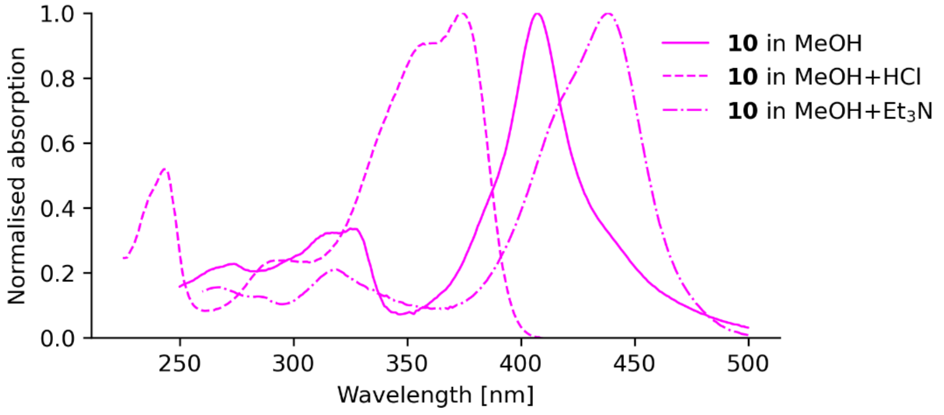

Figure S26: UV-vis spectra of diarylquinocyclopropene **10** in methanol, and with the addition of acid and base. Normalized to λ_{max} in each spectrum.

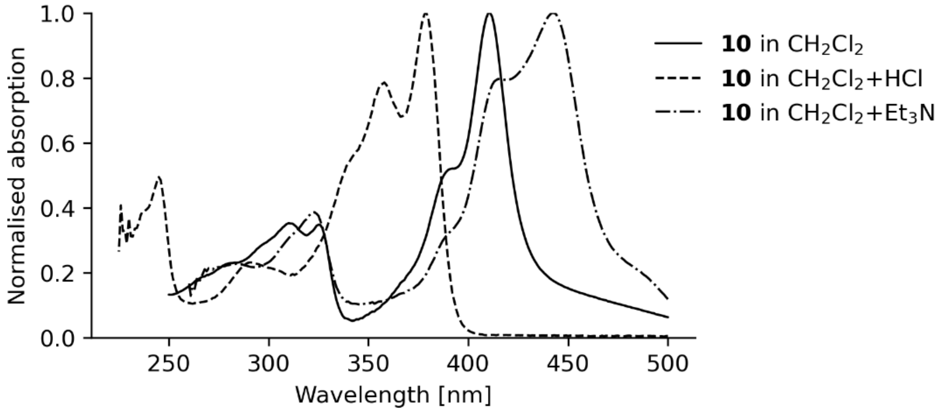

Figure S27: UV-vis spectra of diarylquinocyclopropene **10** in dichloromethane, and with the addition of acid and base. Normalized to λ_{max} in each spectrum.

Table S8: Absorption maxima (nm) of **10** in different solvents, and with the addition of acid and base.

Solvent	Absorption maxima (nm)			
MeCN	278	311	323	408
MeCN + HCl in dioxane	242	290	353	372
MeCN + Et_3N	320	442		
MeOH	274	317	325	407
MeOH + HCl in dioxane	244	296	359	374
MeOH + Et_3N	319	438		
CH_2Cl_2	310	326	385	410
CH_2Cl_2 + HCl in dioxane	245	291	358	379
CH_2Cl_2 + Et_3N	323	442		

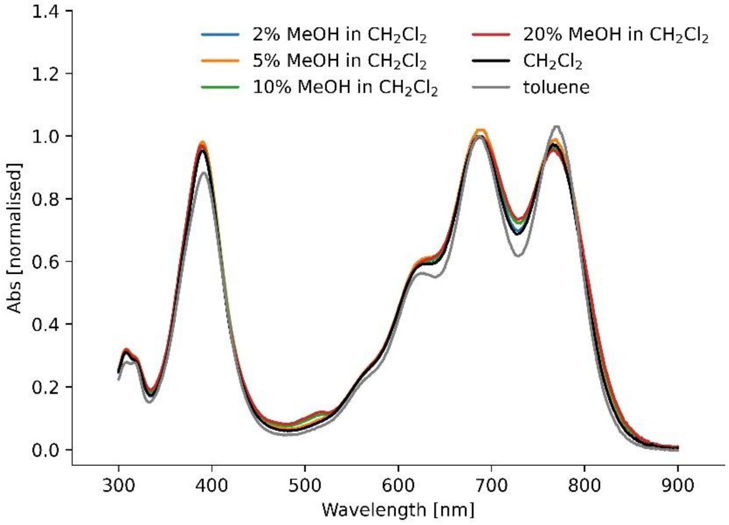

Figure S28: UV-vis spectra of triquino[3]radialene **2** in 2-20% of methanol in CH_2Cl_2, toluene, and CH_2Cl_2. Normalized to λ_{max} in each spectrum.

4.2. Vibrational spectra

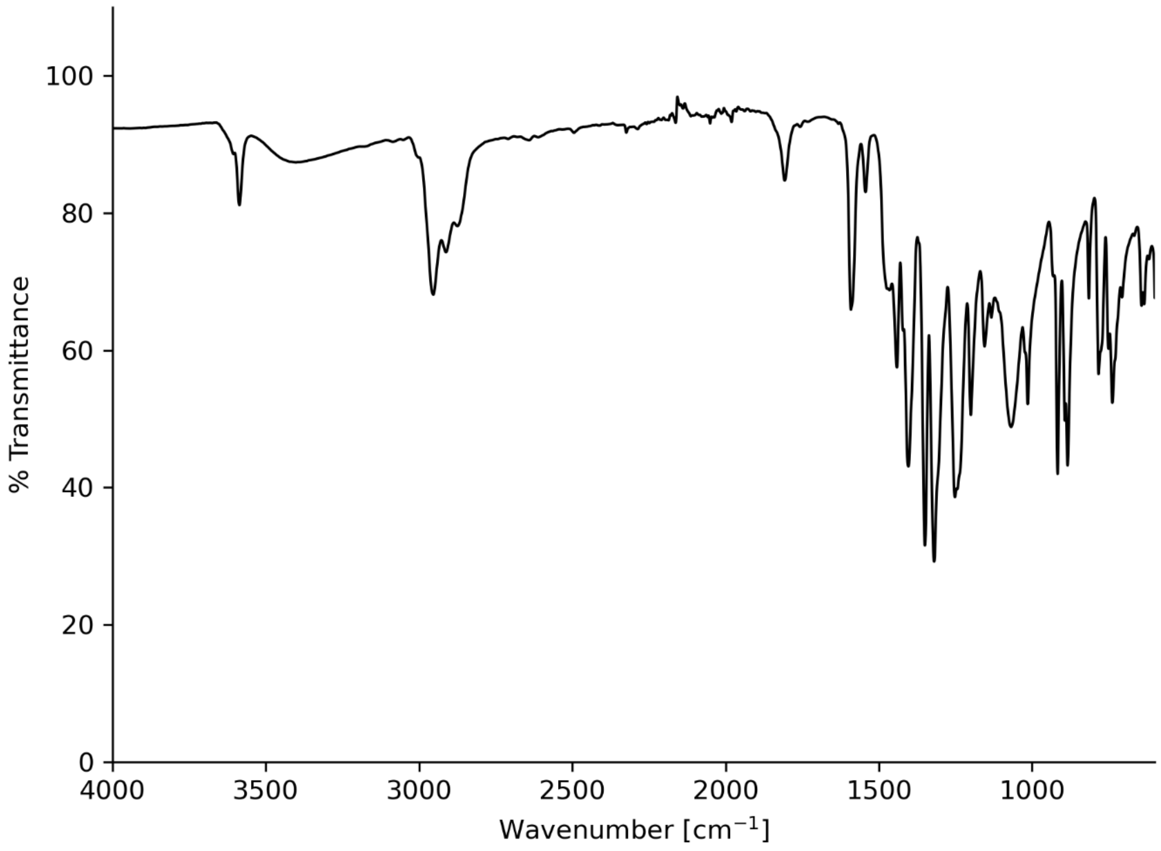

Figure S29: ATR-IR spectrum of a solid sample of diarylquinocyclopropene **10**.

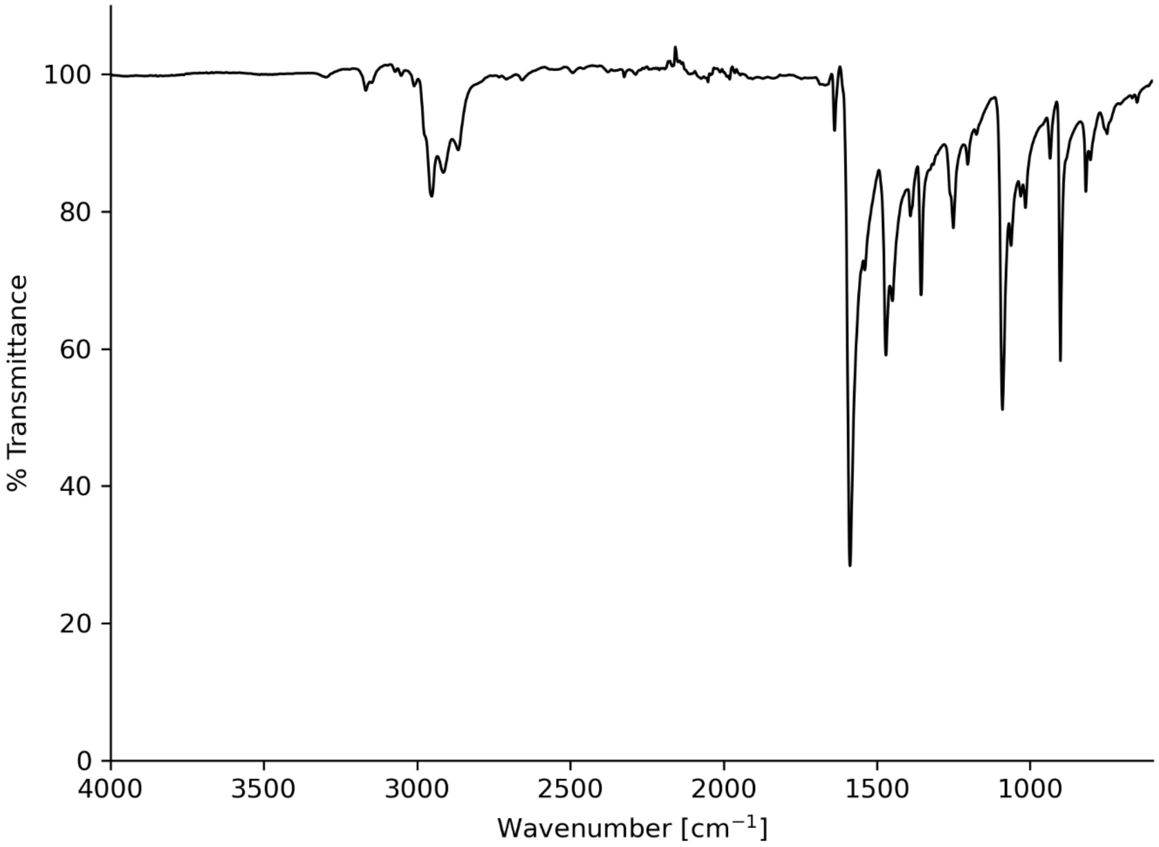

Figure S30: ATR-IR spectrum of a solid sample of triquino[3]radialene **2.** C=O stretch at 1588 cm^{-1}.

4.3. NMR spectra

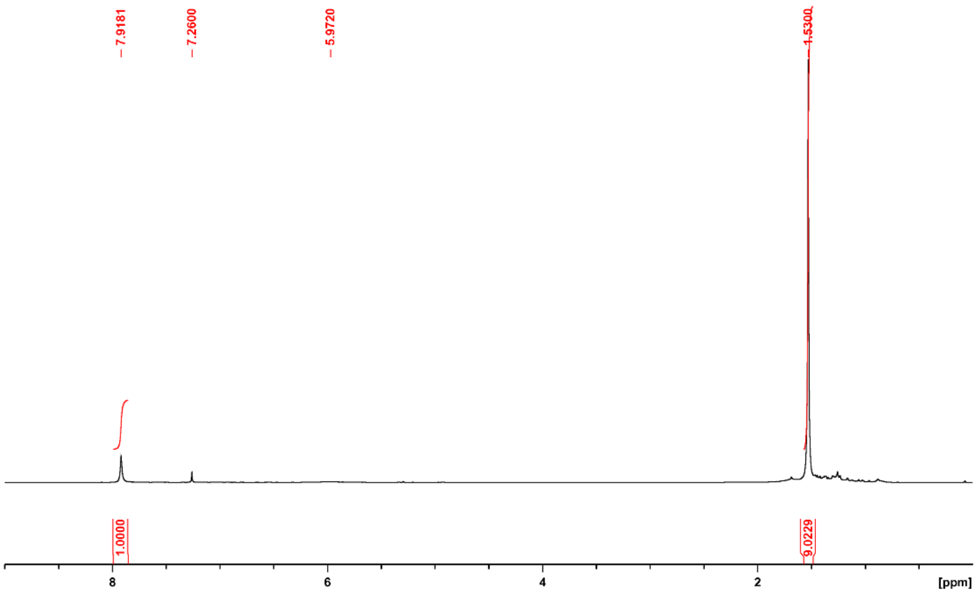

Figure S31: ^{1}H NMR of diarylquinocyclopropene **10** in $CDCl_3$ (400 MHz, 298 K).

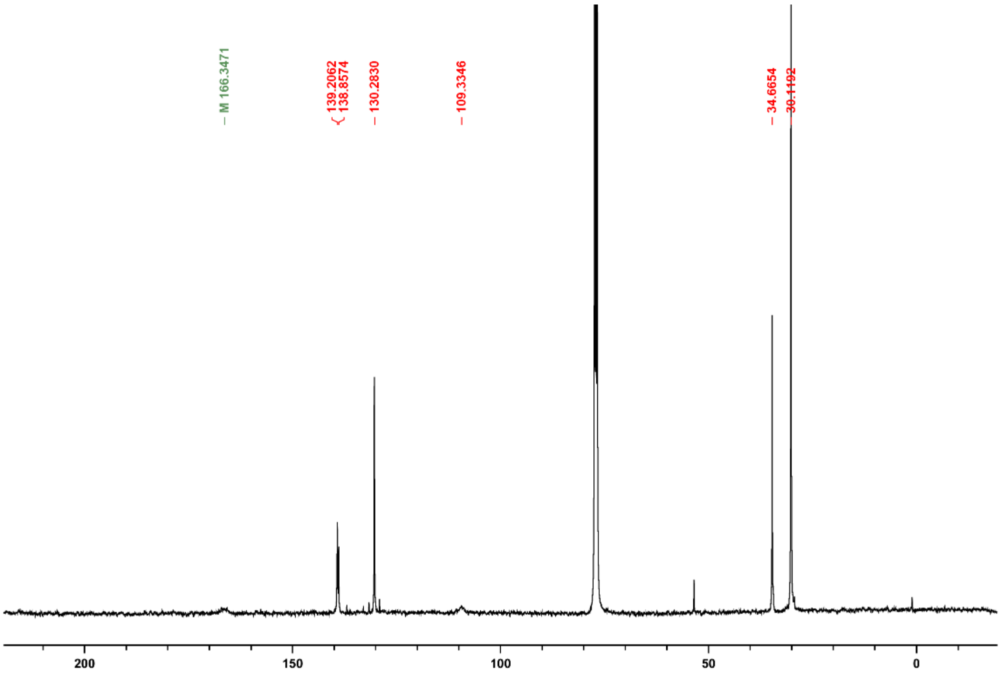

Figure S32: ^{13}C NMR of diarylquinocyclopropene **10** in $CDCl_3$ (100 MHz, 298 K).

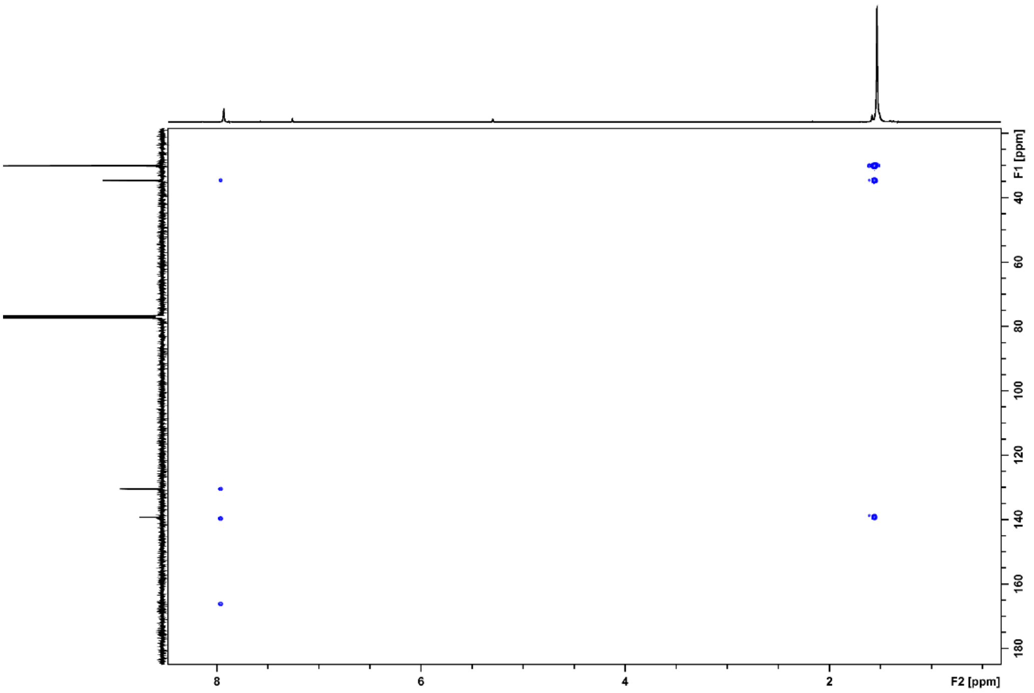

Figure S33: HMBC NMR of diarylquinocyclopropene **10** in $CDCl_3$ (400 MHz, 298 K).

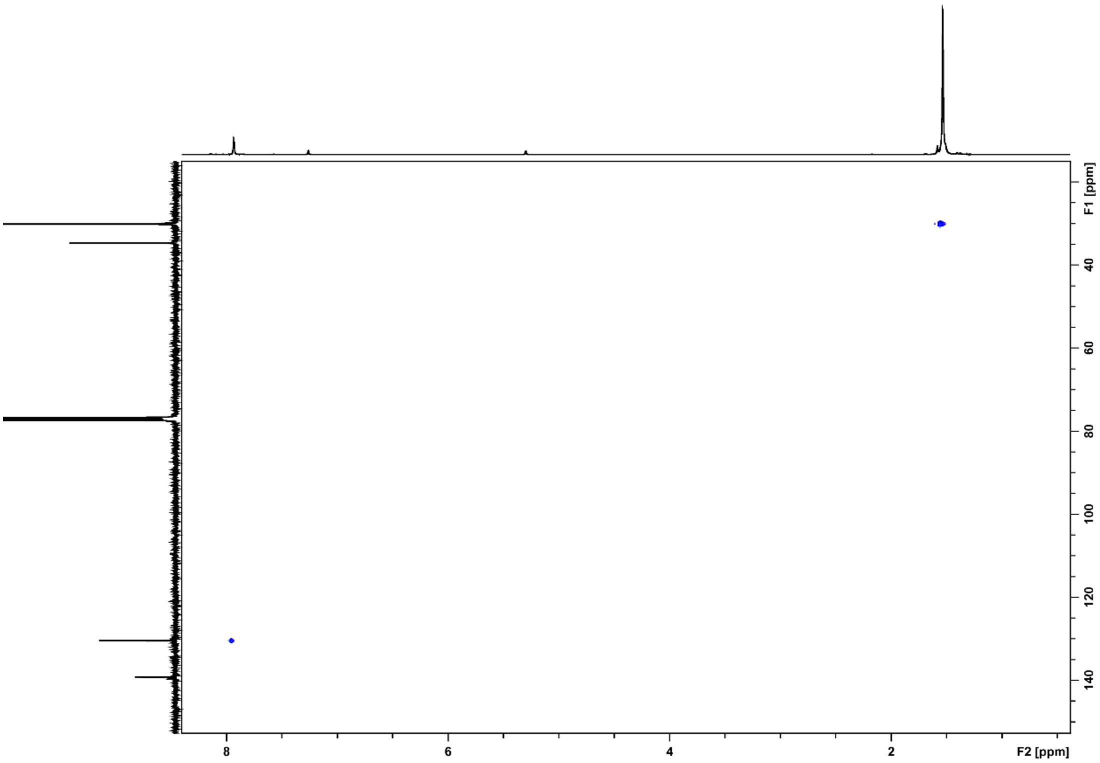

Figure S34: HSQC NMR of diarylquinocyclopropene **10** in $CDCl_3$ (400 MHz, 298 K).

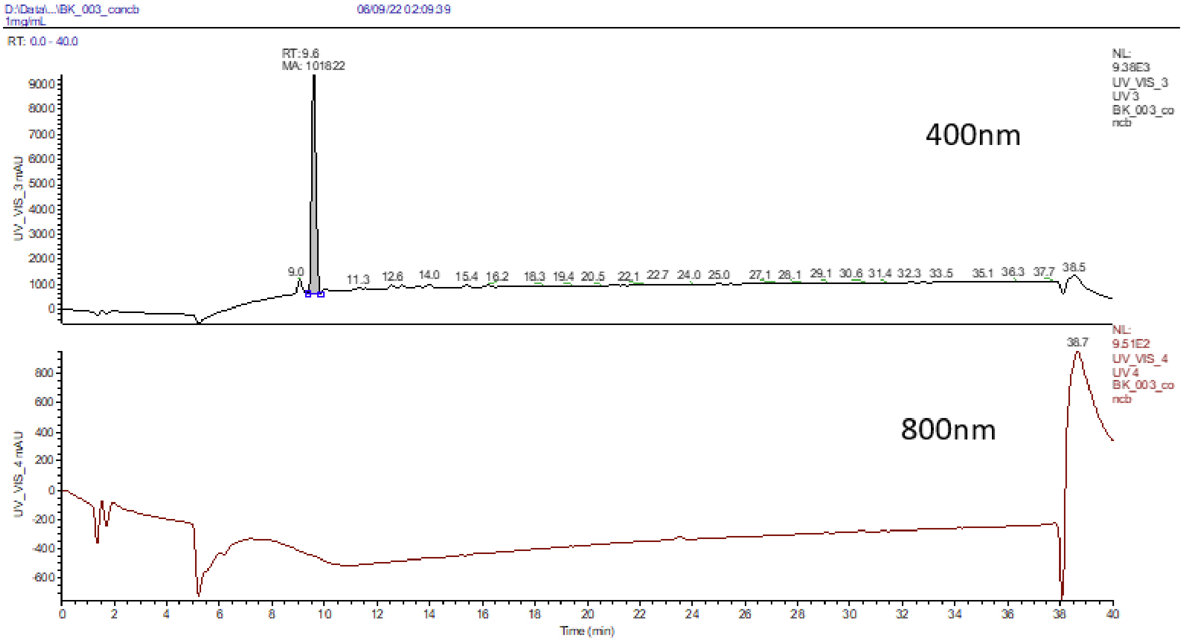

Figure S35: LC-UV-MS of diarylquinocyclopropene **10** in acetonitrile, C8 column, RT 9.8 minutes

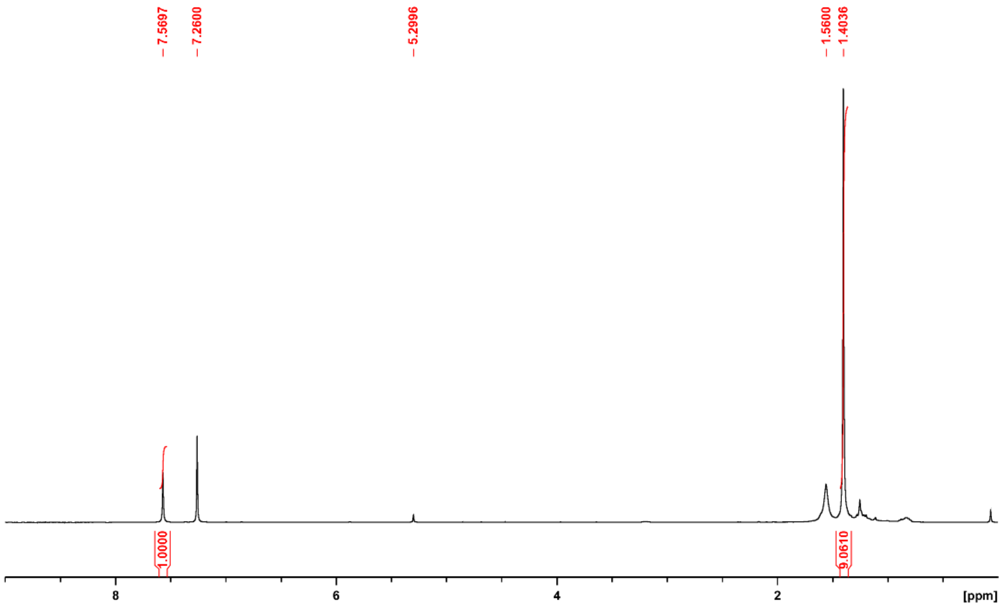

Figure S36: ^{1}H NMR of triquino[3]radialene **2** in $CDCl_3$ (400 MHz, 298 K).

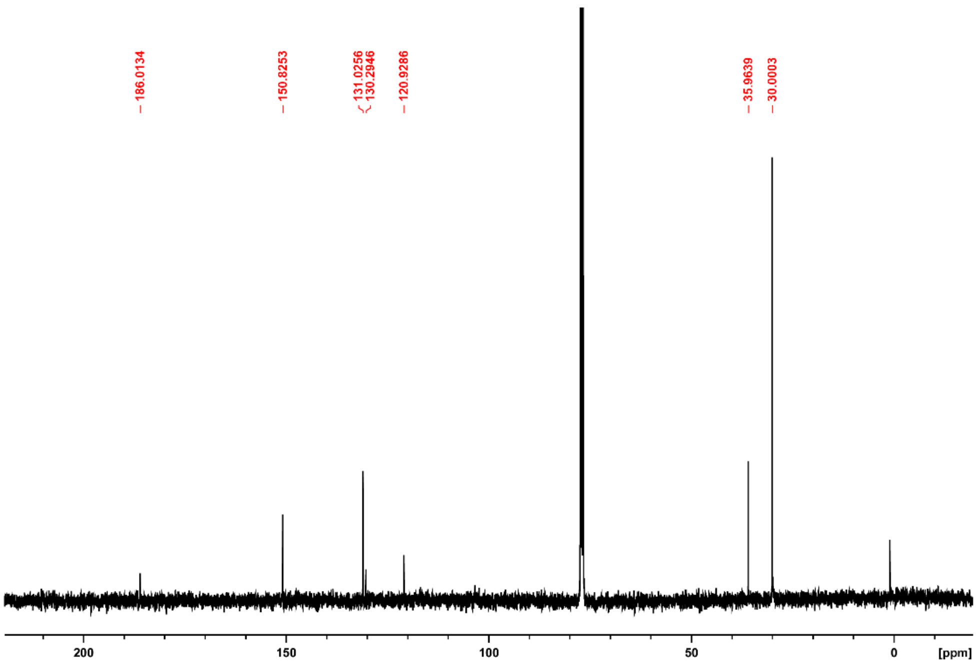

Figure S37: ^{1}H NMR of triquino[3]radialene **2** in $CDCl_3$ (100 MHz, 298 K).

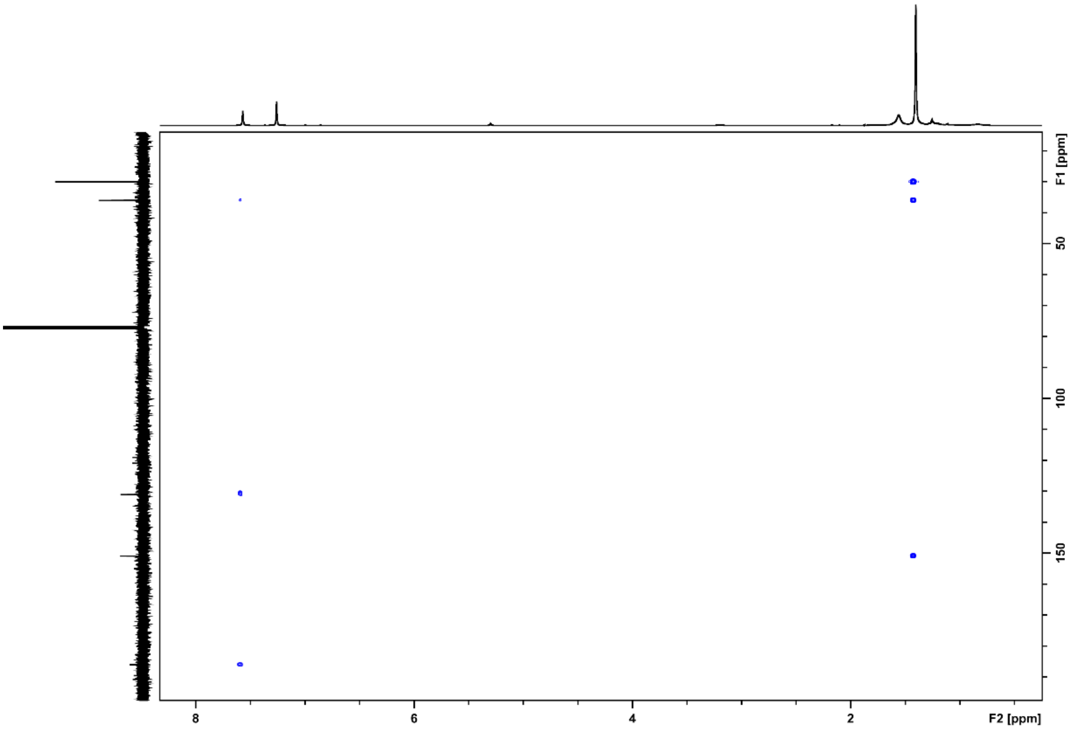

Figure S38: HMBC NMR of triquino[3]radialene **2** in $CDCl_3$ (400 MHz, 298 K).

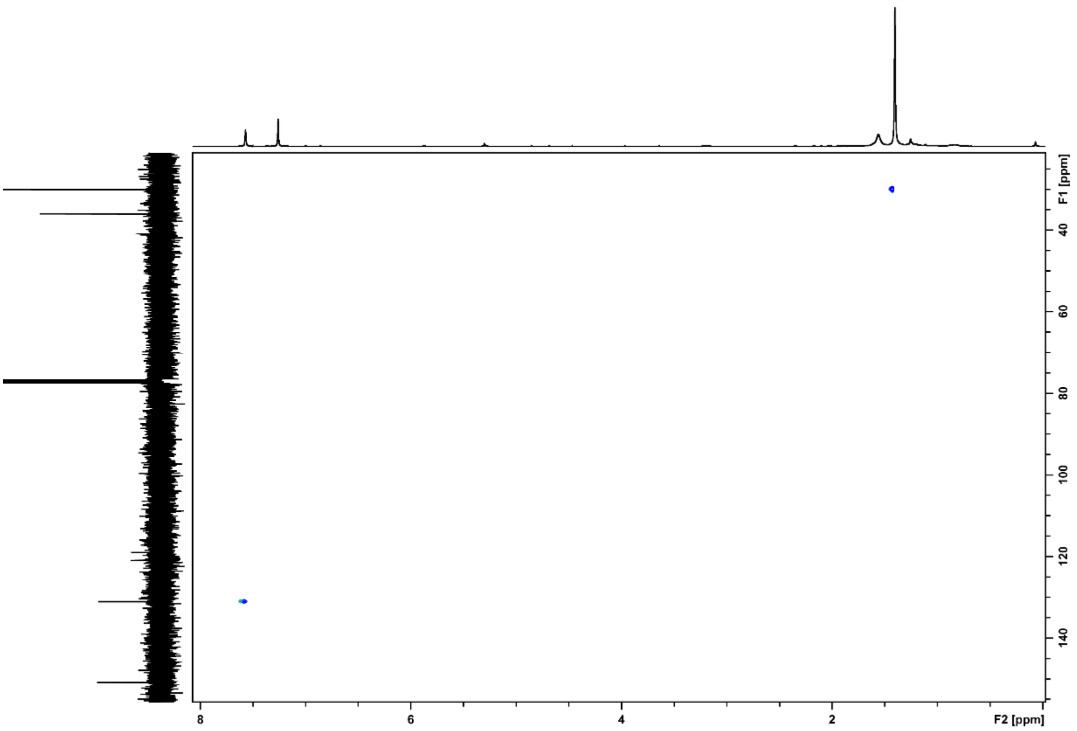

Figure S39: HSQC NMR of triquino[3]radialene **2** in $CDCl_3$ (400 MHz, 298 K).

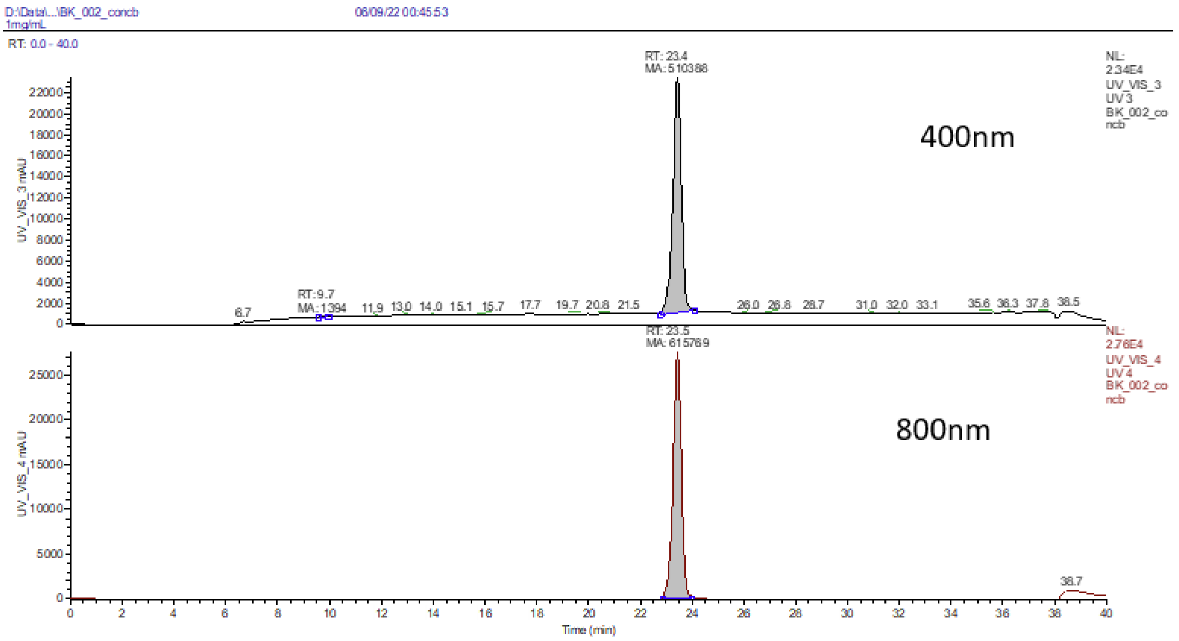

Figure S40: LC-UV-MS of triquino[3]radialene **2** in acetonitrile, C8 column, RT 23.4 minutes.

4.4. X-ray crystallography

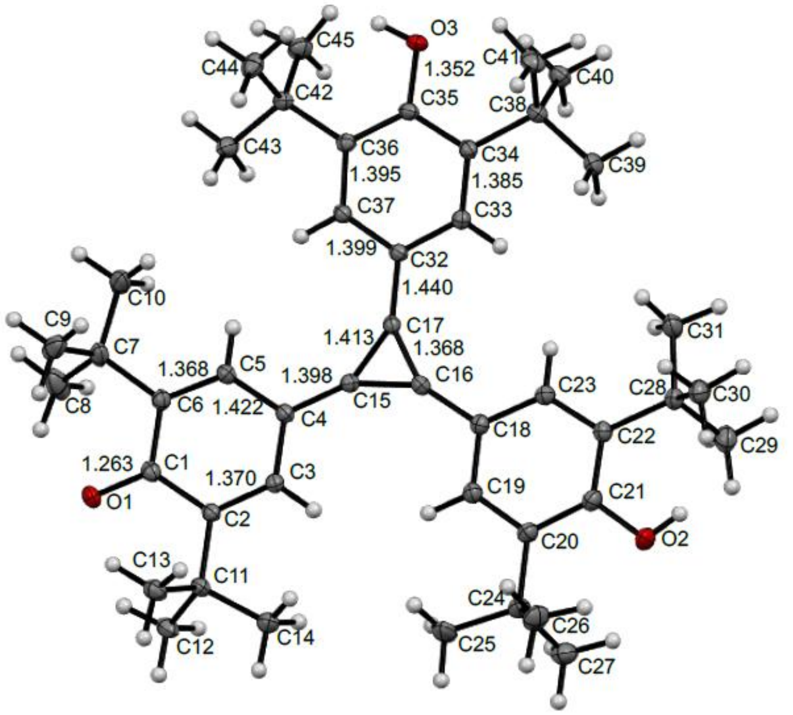

Figure S41: Crystal structure of diarylquinocyclopropene **10** with carbon atoms numbered and select bond-lengths in Å shown.

X-ray crystallography confirms the structural assignment of **10** as a diarylquinocyclopropene. Of the three attached phenol groups, two retain their aromatic structure and the third adopts a quinoidal geometry. The bond lengths between carbon atoms on the quinoidal spoke (C2–C3, and C5–C6) are shorter than bonds between corresponding carbons on the aryl rings (C19–C20, C22–C23, and C33–C34, C36–C37), and similarly the bond between the carbon and oxygen atoms (C1–O1) on the quinoidal ring resembles a carbonyl double bond. Bond lengths are presented in Table S9 below.

Table S9: Carbon-carbon and carbon-oxygen bond lengths of **10**, measured by X-ray crystallography.

atom-atom	Length(stdev) /Å	atom-atom	Length(stdev) /Å
C1–C2	1.469(2)	C22–C23	1.392(2)
C1–C6	1.467(2)	C22–C28	1.538(2)
C2–C3	1.370(2)	C24–C25	1.537(2)
C2–C11	1.537(2)	C24–C26	1.539(2)
C3–C4	1.4296(19)	C24–C27	1.543(2)
C4–C5	1.422(2)	C28–C29	1.544(2)
C4–C15	1.3982(19)	C28–C30	1.543(2)
C5–C6	1.368(2)	C28–C31	1.535(2)
C6–C7	1.536(2)	C32–C33	1.4061(19)
C7–C8	1.534(3)	C32–C37	1.399(2)

C7–C9	1.537(2)	C33–C34	1.384(2)
C7–C10	1.530(2)	C34–C35	1.422(2)
C11–C12	1.540(2)	C34–C38	1.541(2)
C11–C13	1.547(2)	C35–C36	1.412(2)
C11–C14	1.531(2)	C36-C37	1.3947(19)
C15–C16	1.4059(19)	C36–C42	1.5380(19)
C15–C17	1.413(2)	C38–C39	1.533(2)
C16–C17	1.368(2)	C38–C40	1.542(2)
C16–C18	1.4396(19)	C38–C41	1.542(2)
C17–C32	1.4398(19)	C42–C43	1.529(2)
C18–C19	1.399(2)	C42–C44	1.536(2)
C18–C23	1.397(2)	C42–C45	1.545(2)
C19–C20	1.389(2)	O1–C1	1.2627(18)
C20–C21	1.414(2)	O2–C21	1.3665(18)
C20–C24	1.536(2)	O3–C35	1.3519(17)
C21–C22	1.415(2)		

Experimental: Red block-like crystals of **10** were grown by layer recrystallisation from CH_2Cl_2/MeOH. A single crystal with dimensions 0.05×0.05×0.05 mm was mounted on a MicroMount with thin polymer tip and wicking aperture. X-ray diffraction data was collected at 100 K with synchrotron radiation (λ = 0.7107 Å) using the MX1 Macromolecular Crystallography beamline at the Australian Synchrotron.[28] The data collection and integration were performed within the Australian Synchrotron QEGUI and XDS[29] software programs. The crystal structure was solved by intrinsic phasing using SHELXT,[30] followed by successive refinements using full-matrix least-squares method against F^2 with SHELXL[31] contained within Olex2-1.5.[32] All non-hydrogen atoms were refined with anisotropic thermal parameters. Hydrogen atoms were added geometrically and refined using riding thermal parameters. A solvent molecule (dichloromethane) was partially disordered and was modeled over three positions with 0.33 fractional occupancies each. Due to the software set up at Australian Synchrotron, absorption correction and transmission factors are not reported.

Crystal Data for $C_{45}H_{62}O_3$, CH_2Cl_2 (FW = 735.87 g/mol), monoclinic, space group $P2_1/n$ (space group number 14), a = 13.120(3) Å, b = 14.550(3) Å, c = 22.230(4) Å, β = 97.27(3) °, V = 4209.5(15) Å^3, Z = 4, T = 100(2) K, μ = 0.192 mm^{-1}, ρ_{calc} = 1.161 g/cm^3, 84,330 reflections measured (3.354° ≤ 2Θ ≤ 58.56°), 9873 unique (R_{int} = 0.0266, R_{sigma} = 0.0143) which were used in all calculations. The final R_1 was 0.0559 (I > 2σ(I)) and wR_2 was 0.1594 (all data).

5 References

1 R. West and D. C. Zecher, *J. Am. Chem. Soc.*, 1970, **92**, 155–161.
2 R. West, D. C. Zecher and W. Goyert, *J. Am. Chem. Soc.*, 1970, **92**, 149–154.

3 C. Müller, T. Pascher, A. Eriksson, P. Chabera and J. Uhlig, *J. Phys. Chem. A*, 2022, **126**, 4087–4099.
4 S. Grimme, C. Bannwarth and P. Shushkov, *J. Chem. Theory Comput.*, 2017, **13**, 1989–2009.
5 M. J. Frisch, G. W. Trucks, H. B. Schlegel, G. E. Scuseria, M. A. Robb, J. R. Cheeseman, G. Scalmani, V. Barone, G. A. Petersson, H. Nakatsuji, X. Li, M. Caricato, A. V. Marenich, J. Bloino, B. G. Janesko, R. Gomperts, B. Mennucci, H. P. Hratchian, J. V. Ortiz, A. F. Izmaylov, J. L. Sonnenberg, D. Williams-Young, F. Ding, F. Lipparini, F. Egidi, J. Goings, B. Peng, A. Petrone, T. Henderson, D. Ranasinghe, V. G. Zakrzewski, J. Gao, N. Rega, G. Zheng, W. Liang, M. Hada, M. Ehara, K. Toyota, R. Fukuda, J. Hasegawa, M. Ishida, T. Nakajima, Y. Honda, O. Kitao, H. Nakai, T. Vreven, K. Throssell, J. A. Montgomery Jr., J. E. Peralta, F. Ogliaro, M. J. Bearpark, J. J. Heyd, E. N. Brothers, K. N. Kudin, V. N. Staroverov, T. A. Keith, R. Kobayashi, J. Normand, K. Raghavachari, A. P. Rendell, J. C. Burant, S. S. Iyengar, J. Tomasi, M. Cossi, J. M. Millam, M. Klene, C. Adamo, R. Cammi, J. W. Ochterski, R. L. Martin, K. Morokuma, O. Farkas, J. B. Foresman and D. J. Fox, *Gaussian 16 Revision A.03*, Wallingford CT, 2016.
6 T. Yanai, D. P. Tew and N. C. Handy, *Chem. Phys. Lett.*, 2004, **393**, 51–57.
7 R. Ditchfield, W. J. Hehre and J. A. Pople, *J. Chem. Phys.*, 1971, **54**, 724–728.
8 W. J. Hehre, R. Ditchfield and J. A. Pople, *J. Chem. Phys.*, 1972, **56**, 2257–2261.
9 P. C. Hariharan and J. A. Pople, *Theor. Chim. Acta*, 1973, **28**, 213–222.
10 A. D. Becke, *J. Chem. Phys.*, 1993, **98**, 5648.
11 P. J. Stephens, F. J. Devlin, C. F. Chabalowski and M. J. Frisch, *J. Phys. Chem.*, 1994, **98**, 11623–11627.
12 H.-J. Werner, P. J. Knowles, G. Knizia, F. R. Manby and M. Schütz, *WIREs Comput Mol Sci*, 2012, **2**, 242–253.
13 H.-J. Werner, P. J. Knowles, F. R. Manby, J. A. Black, K. Doll, A. Heßelmann, D. Kats, A. Köhn, T. Korona, D. A. Kreplin, Q. Ma, T. F. Miller III, A. Mitrushchenkov, K. A. Peterson, I. Polyak, G. Rauhut and M. Sibaev, *J. Chem. Phys.*, 2020, **152**, 144107.
14 H.-J. Werner, P. J. Knowles, P. Celani, W. Györffy, A. Hesselmann, D. Kats, G. Knizia, A. Köhn, T. Korona, D. Kreplin, R. Lindh, Q. Ma, F. R. Manby, A. Mitrushenkov, G. Rauhut, M. Schütz, K. R. Shamasundar, T. B. Adler, R. D. Amos, S. J. Bennie, A. Bernhardsson, A. Berning, J. A. Black, P. J. Bygrave, R. Cimiraglia, D. L. Cooper, D. Coughtrie, M. J. O. Deegan, A. J. Dobbyn, K. Doll, M. Dornbach, F. Eckert, S. Erfort, E. Goll, C. Hampel, G. Hetzer, J. G. Hill, M. Hodges, T. Hrenar, G. Jansen, C. Köppl, C. Kollmar, S. J. R. Lee, Y. Liu, A. W. Lloyd, R. A. Mata, A. J. May, B. Mussard, S. J. McNicholas, W. Meyer, T. F. M. III, M. E. Mura, A. Nicklass, D. P. O'Neill, P. Palmieri, D. Peng, K. A. Peterson, K. Pflüger, R. Pitzer, I. Polyak, M. Reiher, J. O. Richardson, J. B. Robinson, B. Schröder, M. Schwilk, T. Shiozaki, M. Sibaev, H. Stoll, A. J. Stone, R. Tarroni, T. Thorsteinsson, J. Toulouse, M. Wang, M. Welborn and B. Ziegler, MOLPRO, 2020.1, a package of ab initio programs.
15 T. H. Dunning Jr., *J. Chem. Phys.*, 1989, **90**, 1007–1023.
16 P. Celani, H. Stoll, H.-J. Werner and P. J. Knowles *, *Mol. Phys.*, 2004, **102**, 2369–2379.
17 E. Matito, M. Duran and M. Solà, *J. Chem. Phys.*, 2004, **122**, 014109.
18 J. Poater, X. Fradera, M. Duran and M. Solà, *Chem. – Eur. J.*, 2003, **9**, 400–406.
19 M. Giambiagi, M. S. de Giambiagi, C. D. dos S. Silva and A. P. de Figueiredo, *Phys. Chem. Chem. Phys.*, 2000, **2**, 3381–3392.
20 P. Bultinck, R. Ponec and S. Van Damme, *J. Phys. Org. Chem.*, 2005, **18**, 706–718.
21 T. A. Keith, AIMAll (version Version 19.10.12) TK Gristmill Software, Overland Park KS, USA 2019.
22 Matito, E., ESI-3D: Electron Sharing Indices Program for 3D Molecular Space Partitioning http://iqc.udg.es/eduard/ESI 2006.
23 E. Matito, M. Solà, P. Salvador and M. Duran, *Faraday Discuss.*, 2007, **135**, 325–345.

24 N. Proos Vedin, S. Escayola, S. Radenković, M. Solà and H. Ottosson, *J. Phys. Chem. A*, 2024, **128**, 4493–4506.
25 R. Herges and D. Geuenich, *J. Phys. Chem. A*, 2001, **105**, 3214–3220.
26 D. Geuenich, K. Hess, F. Köhler and R. Herges, *Chem. Rev.*, 2005, **105**, 3758–3772.
27 E. D. Glendening, J. K. Badenhoop, A. E. Reed, J. E. Carpenter, J. A. Bohmann, C. M. Morales, P. Karafiloglou, C. R. Landis and F. Weinhold, NBO 7.0 Theoretical Chemistry Institute, University of Wisconsin, Madison 2018.
28 T. M. McPhillips, S. E. McPhillips, H.-J. Chiu, A. E. Cohen, A. M. Deacon, P. J. Ellis, E. Garman, A. Gonzalez, N. K. Sauter, R. P. Phizackerley, S. M. Soltis and P. Kuhn, *J. Synchrotron Radiat.*, 2002, **9**, 401–406.
29 W. Kabsch, *Acta Crystallogr. D Biol. Crystallogr.*, 2010, **66**, 125–132.
30 G. M. Sheldrick, *Acta Crystallogr. Sect. Found. Adv.*, 2015, **71**, 3–8.
31 G. M. Sheldrick, *Acta Crystallogr. Sect. C Struct. Chem.*, 2015, **71**, 3–8.
32 O. V. Dolomanov, L. J. Bourhis, R. J. Gildea, J. a. K. Howard and H. Puschmann, *J. Appl. Crystallogr.*, 2009, **42**, 339–341.
